# Ensuring a Successful Transition From Cytology to Human Papillomavirus–Based Primary Cervical Cancer Screening in Canada by Investigating the Psychosocial Correlates of Women’s Intentions: Protocol for an Observational Study

**DOI:** 10.2196/38917

**Published:** 2022-06-16

**Authors:** Gabrielle Griffin-Mathieu, Ben Haward, Ovidiu Tatar, Patricia Zhu, Samara Perez, Gilla K Shapiro, Emily McBride, Erika L Thompson, Laurie W Smith, Aisha K Lofters, Ellen M Daley, Juliet R Guichon, Jo Waller, Marc Steben, Kathleen M Decker, Marie-Helene Mayrand, Julia M L Brotherton, Gina S Ogilvie, Gregory D Zimet, Teresa Norris, Zeev Rosberger

**Affiliations:** 1 Lady Davis Institute for Medical Research McGill University Jewish General Hospital Montreal, QC Canada; 2 Research Center Centre Hospitalier de l'Université de Montreal Montreal, QC Canada; 3 Department of Psychiatry McGill University Montreal, QC Canada; 4 Cedars Cancer Center McGill University Health Centre Montreal, QC Canada; 5 Department of Oncology McGill University Montreal, QC Canada; 6 HPV Global Action Montreal, QC Canada; 7 Department of Supportive Care Princess Margaret Cancer Centre University Health Network Toronto, ON Canada; 8 Department of Behavioural Science & Health University College London London United Kingdom; 9 Department of Biostatistics and Epidemiology, School of Public Health The University of North Texas Health Science Center Fort Worth, TX United States; 10 BC Cancer Agency Vancouver, BC Canada; 11 Department of Family and Community Medicine University of Toronto Toronto, ON Canada; 12 Department of Family and Community Medicine Women’s College Hospital Toronto, ON Canada; 13 College of Public Health University of South Florida Tampa, FL United States; 14 Department of Community Health Sciences University of Calgary Calgary, AB Canada; 15 School of Cancer and Pharmaceutical Sciences King’s College London London United Kingdom; 16 School of Public Health Université de Montréal Montreal, QC Canada; 17 Department of Community Health Sciences University of Manitoba Winnipeg, MB Canada; 18 Cancer Care Manitoba Research Institute Winnipeg, MB Canada; 19 Département d'obstétrique-gynécologie Université de Montreal Montreal, QC Canada; 20 Melbourne School of Population and Global Health University of Melbourne Melbourne Australia; 21 Population Health Australian Centre for the Prevention of Cervical Cancer Melbourne Australia; 22 Faculty of Medicine University of British Columbia Vancouver, BC Canada; 23 BC Women’s Hospital Vancouver, BC Canada; 24 School of Medicine Indiana University Bloomington, IN United States; 25 Department of Psychology McGill University Montreal, QC Canada

**Keywords:** human papillomavirus, HPV-based primary screening, cervical cancer, cervical cancer screening, cancer prevention, knowledge, attitudes and beliefs, preferences, HPV test acceptability, HPV self-sampling, Pap testing, cytology, mobile phone

## Abstract

**Background:**

The human papillomavirus (HPV) test has emerged as a significant improvement over cytology for primary cervical cancer screening. In Canada, provinces and territories are moving toward implementing HPV testing in cervical cancer screening programs. Although an abundance of research exists on the benefits of HPV-based screening, there is a dearth of research examining women’s understanding of HPV testing. In other countries, failure to adequately address women’s concerns about changes has disrupted the implementation of HPV-based screening.

**Objective:**

The aims of the multipart study described in this paper are to develop psychometrically valid measures of cervical cancer screening–related knowledge, attitudes, and beliefs; to examine the feasibility of a questionnaire examining psychosocial factors related to HPV-based screening; and to investigate psychosocial correlates of women’s intentions to participate in HPV-based screening.

**Methods:**

We conducted a web-based survey (study 1) of Canadian women to assess the acceptability and feasibility of a questionnaire, including the validation of scales examining cervical cancer knowledge, HPV testing knowledge, HPV testing attitudes and beliefs, and HPV test self-sampling attitudes and beliefs. Preferences for cervical cancer screening were assessed using the best-worst scaling methodology. A second web-based survey (study 2) will be administered to a national sample of Canadian women between June 2022 and July 2022 using the validated scales. Differences in the knowledge, attitudes, beliefs, and preferences of women who are currently either underscreened or adequately screened for cervical cancer will be examined through bivariate analyses. Multinomial logistic regression will be used to estimate the associations between psychosocial and sociodemographic factors and intentions to undergo HPV-based screening.

**Results:**

Between October 2021 and November 2021, a total of 1230 participants completed the questionnaire in study 1, and 1027 (83.49%) responses were retained after data cleaning methods were applied. Feasibility was comparable with similar population-based surveys in terms of survey length, participant attrition, and the number of participants excluded after data cleaning. As of May 2022, analysis of study 1 is ongoing, and results are expected to be published in the summer of 2022. Data collection is expected to begin for study 2 in the summer of 2022. Results are expected to be published between late 2022 and early 2023.

**Conclusions:**

Findings will provide direction for Canadian public health authorities to align guidelines to address women’s concerns and optimize the acceptability and uptake of HPV-based primary screening. Validated scales can be used by other researchers to improve and standardize the measurement of psychosocial factors affecting HPV test acceptability. Study results will be disseminated through peer-reviewed journal articles; conference presentations; and direct communication with researchers, clinicians, policy makers, media, and specialty organizations.

**International Registered Report Identifier (IRRID):**

DERR1-10.2196/38917

## Introduction

### Background

Cervical cancer is the fourth most common cancer in women and presents a significant risk to all people with a cervix [1-3]. In 2018, an estimated 570,000 women were newly diagnosed with cervical cancer worldwide, with 311,000 deaths from the disease [4]. In Canada, >1300 women are diagnosed with and >400 women die from cervical cancer each year [5]. Cytology testing using the Papanicolaou smear, commonly referred to as the Pap test, allows for the detection and subsequent treatment of precancerous lesions that may lead to cervical cancer. Canadian women have been widely screened for cervical cancer using the Pap test for >50 years [6]. There are guidelines in each province and territory that currently recommend screening every 1 to 3 years, starting at the age of 21 or 25 years [7], and most Canadian jurisdictions have organized screening programs [8].

Cervical cancer is caused by persistent infection with high-risk human papillomavirus (HPV) types [9]. The HPV test, which detects high-risk HPV DNA in cervical cells, has emerged as a significant improvement over cytology for cervical cancer screening. Compared with cytology-based screening, HPV-based screening has been shown to offer 60% to 70% higher protection against the development of cervical cancer [10,11]. The HPV test shows improved sensitivity, and negative test results have high negative predictive value, thus providing greater reassurance against cervical lesion development [12]. Furthermore, this allows for increased intervals between cervical screenings and reduced testing costs [12]. HPV testing also introduces the possibility for vaginal self-sampling by the patient. This is not possible with cytology, which requires cervical cell collection by a health care provider, a process that can be uncomfortable or invasive [13,14]. Meta-analyses have shown that self-sampling has similar test accuracy when compared with health care provider–administered sampling [15] and could increase uptake among those who are underscreened when provided as a screening option [15,16].

Therefore, currently, HPV testing is considered the preferred method of screening by the World Health Organization [17] and is recommended by multiple specialty organizations worldwide (eg, United States Preventive Services Task Force [18], European Society of Gynaecologic Oncology, and European Federation of Colposcopy [19]). Several countries have implemented HPV-based organized screening programs (eg, Australia, the United Kingdom, and the Netherlands), including the use of self-sampling as a collection option [20,21]. However, program implementations have encountered challenges. For example, in Australia, before the introduction of HPV testing, a web-based petition against the proposed changes gained widespread support. In an analysis of comments on the petition, Australian women felt that the policy *devalued or threatened women’s health* and represented a government cost-cutting measure and that increased screening intervals and later age of screening initiation would prevent early detection of cervical abnormalities [22]. Similarly, in Wales, increase in screening intervals associated with the shift to HPV-based screening has been met with public backlash, with >1.2 million signatures on a web-based petition against the change at the time of writing [23]. Although these guidelines are grounded in evidence, a disconnect has been observed between women’s and public health authorities’ views on cervical cancer screening changes. These health authorities failed to effectively and proactively communicate why changes were warranted, why screening intervals may change, and what HPV test results indicate [22-26].

### Screening Landscape in Canada

In Canada, provinces and territories are in different planning phases for HPV-based cervical cancer screening programs [7,27], and the nationwide introduction of HPV-based primary screening is a key priority of the recent Canadian Partnership Against Cancer (CPAC) Action Plan for the Elimination of Cervical Cancer [5]. Although cytology-based screening is well established in Canada, screening coverage has failed to reach the CPAC target of including ≥80% of women [28]. This suggests that innovative approaches are needed to reach those women who are underscreened (ie, longer than 3 years since their previous Pap test or never screened). Approximately 40% of women in Canada diagnosed with cervical cancer report either never having had a Pap test or not having had one in >3 years [29]. Underscreened women are often members of ethnic, linguistic, gender, and sexual minorities or have lower socioeconomic status [30-33]. In Canada, only 65% of recent female immigrants reported having a Pap test in the previous 3 years, and 26% of non-English or French speakers reported never having a Pap test [29]. In a study of cancer screening registries in Ontario, transgender patients were significantly less likely than cisgender patients to be screened for cervical cancer (56% vs 72%) [32], reflective of barriers faced by transgender and other gender-diverse people in seeking cervical cancer screening [2]. A study by Decker et al [34] comparing screening rates and outcomes between First Nations women and all other women in Manitoba suggested lower screening rates among First Nations women aged ≥40 years and significantly higher rates of cervical lesions and cancers in First Nations women overall. Targeted solutions must consider these factors. For underscreened women, the introduction of HPV self-sampling could be important for ensuring that cervical cancer screening is more accessible and acceptable [13-15,35,36]. A meta-analysis of 37 studies by Nelson et al [37] found that women who used and indicated acceptability of self-sampling for future screening did so because of its convenience, privacy, and ease of use. However, identified barriers include women’s lack of confidence in their ability to correctly collect the specimen, lack of confidence in the test result, and discomfort with the procedure [37,38].

The concerns of adequately screened women (ie, <3 years since their previous Pap test) must also be addressed. Clear and open dialogue with these women is needed to prevent confusion and provide reassurance that the switch to HPV-based screening is evidence-based and represents an improvement over cytology-based screening. Currently, there is a dearth of research examining Canadian women’s perceptions and understanding of HPV-based screening and potential changes to screening guidelines. To avoid problems that may arise in Canada as program implementations advance, efforts should be made to understand and address the concerns of women.

### Measuring Knowledge, Attitudes, and Beliefs About Cervical Cancer Screening

From both theoretical and practical perspectives, knowledge is a determinant of engagement in protective health behaviors [24,39,40]. In a systematic review of psychosocial factors associated with intentions and acceptability of HPV testing, knowledge was associated with greater acceptability of screening using the HPV test [24]. In Canada, poor cervical cancer screening knowledge has been identified as a key barrier to screening in populations such as immigrant women and ethnic minority communities [41-43]. In a recent systematic review, specific attitudes and beliefs about HPV testing have been identified as both barriers to and facilitators of HPV test acceptability [24]. For example, high perceived benefits of the HPV test were associated with greater HPV test acceptability, whereas negative emotions (eg, shame associated with testing for a sexually transmitted infection) related to HPV testing were associated with lower HPV test acceptability. Importantly, these findings and many additional attitudes and beliefs (eg, high perceived susceptibility to cervical cancer, negative perceived emotional reaction to HPV test results, and high perceived severity of HPV infection) have not been studied and confirmed in Canada-wide samples.

Several measures exist for cervical cancer knowledge [44-47], a subscale has been developed for measuring knowledge of HPV testing [48], and 2 measures of HPV testing and self-sampling attitudes and beliefs have been developed [49,50]. However, existing measures have shown insufficient psychometric testing or suboptimal psychometric properties. In addition, in a fast-moving field, many measures are not up-to-date with evidence from the literature on cervical cancer and HPV testing or do not include other important factors associated with HPV test acceptability (eg, negative emotions related to HPV testing [24]). As women’s perceptions of cervical cancer and HPV testing are multifaceted, comprehensive and valid measurement tools are crucial for identifying attitudes, beliefs, and knowledge gaps that could be barriers to the acceptance and uptake of HPV-based screening.

### Measuring Preferences Regarding Cervical Cancer Screening

Preferences refer to a distinct form of attitude that provides information about the relative value and ranking an individual assigns to certain options over others. Measuring preferences is important in the context of cervical cancer screening, as multiple approaches (eg, varying screening intervals and use of self-sampling) are being considered for implementation [5]. Understanding Canadian women’s preferences can provide insight into the acceptability of different screening options and illuminate any potential disconnect between women and public health regarding the implementation of HPV-based screening programs. In particular, by examining women’s preferences for screening intervals and age of screening initiation, 2 major points of contention observed in other program implementations [22,51,52], public health can optimize communications to address concerns and ensure acceptability of HPV-based screening guidelines [24]. Furthermore, examining the preferences of underscreened women and adequately screened women separately can help to inform targeted communications for these groups.

### This Study

The proposed study will use a multistep approach to examine women’s knowledge, attitudes, beliefs, and preferences toward HPV testing and self-sampling. A preparatory study (study 1) was focused on the development and psychometric validation of scales measuring cervical cancer screening–related knowledge, attitudes, and beliefs. In addition, given the challenges of investigating women’s views toward a screening approach that has not yet been implemented, this study examined the feasibility and structure of a survey that examines these factors in Canadian women. Results of study 1 will yield validated scales to administer to a large sample of Canadian women (study 2) to estimate the associations between psychosocial factors and women’s intentions to participate in HPV-based screening programs. Furthermore, study 2 will survey and compare differences in psychosocial factors and HPV test intentions among adequately screened women and underscreened women, providing timely data to address emerging challenges in both groups.

### Objectives

The main objectives of study 1 (scale and questionnaire development) are as follows:

1. To develop psychometrically valid scales to assess women’s knowledge of cervical cancer and attitudes, beliefs, and knowledge about HPV testing and self-sampling.
2. To evaluate the feasibility and acceptability of using a web-based survey related to Canadian women’s knowledge, attitudes, beliefs, and preferences about new HPV testing–based cervical cancer screening programs.

The main objectives of study 2 (expanded population-based survey of Canadian women) are as follows:

1. To estimate differences in HPV and cervical cancer screening knowledge and attitudes, beliefs, and preferences regarding HPV testing between women who are adequately screened and those who are underscreened.
2. To estimate the multivariable associations between psychosocial factors (eg, knowledge, attitudes, beliefs, and sociodemographics) and intentions to use HPV testing and self-sampling in women who are adequately screened and those who are underscreened.

## Study 1: Questionnaire Development and Scale Validation

### Methods

#### Study 1: Ethics Approval

Study 1 received approval from the Research Ethics Board of the Centre intégré universitaire de santé et de services sociaux (the Integrated Health and Social Services University Network) of West-Central Montreal (project ID 2021-2632) in March 2021.

#### Theoretical Frameworks

Questionnaire development and item selection for the development of the scales were guided by relevant theoretical frameworks. The selection of psychosocial factors influencing intentions to engage in HPV testing was informed by 2 theories: the Theory of Planned Behavior (TPB), which suggests that attitudes, beliefs, subjective norms, and perceived behavioral control influence intentions and subsequent health behaviors [53], and the Health Belief Model (HBM), which posits that the likelihood of behavior change is influenced by perceived susceptibility, seriousness and threat of the disease, perceived benefits of adopting protective health behaviors (ie, screening), cues to action (eg, information and social influence), sociodemographics, and knowledge [54].

#### Study Design

To build robust measures, we followed a rigorous stepwise process involving review of the scientific literature, discussions, and consensus development with our experienced team of researchers and consultation with project collaborators and the population of interest. This process is described chronologically, separated into 3 steps: phase 1A—questionnaire development, phase 1B—questionnaire validation, and phase 1C—survey testing and psychometric validation. As of May 2022, phases 1A and 1B are completed, and data analysis is ongoing for phase 1C.

#### Phase 1A: Questionnaire Development

##### Literature Search

We reviewed the literature for existing relevant scales using a validated and updated search strategy that we used for a recently published knowledge synthesis summarizing factors associated with HPV test acceptability in primary screening for cervical cancer [24]. Embase, Global Health, PsycINFO, MEDLINE, and CINAHL databases were searched from January 2017 to October 2019. After removing duplicates, a total of 1477 references were screened by title and abstract, and 89 full-text articles were reviewed to identify relevant scales. Our team found and reviewed 13 scales (including 4 scales that were identified in past literature searches [44,45,48-50,55-62]). Our overall conclusions indicated that existing questionnaires were incomplete in the following ways: not including questions about HPV testing in cervical cancer screening (eg, only including items related to the Pap test), having inadequate psychometric validation analyses (eg, no factor analysis, only partial factor analysis with exploratory factor analysis [EFA], or no reliability testing at all), having inadequate psychometric properties (eg, internal consistency <0.6), being unsuitable for our study design (eg, being designed to administer verbally), and having limited sampling characteristics (eg, only adolescents or in a specific culture or context). Nevertheless, many individual items were potentially relevant to our objectives.

##### Item Selection

**Figure 1:**
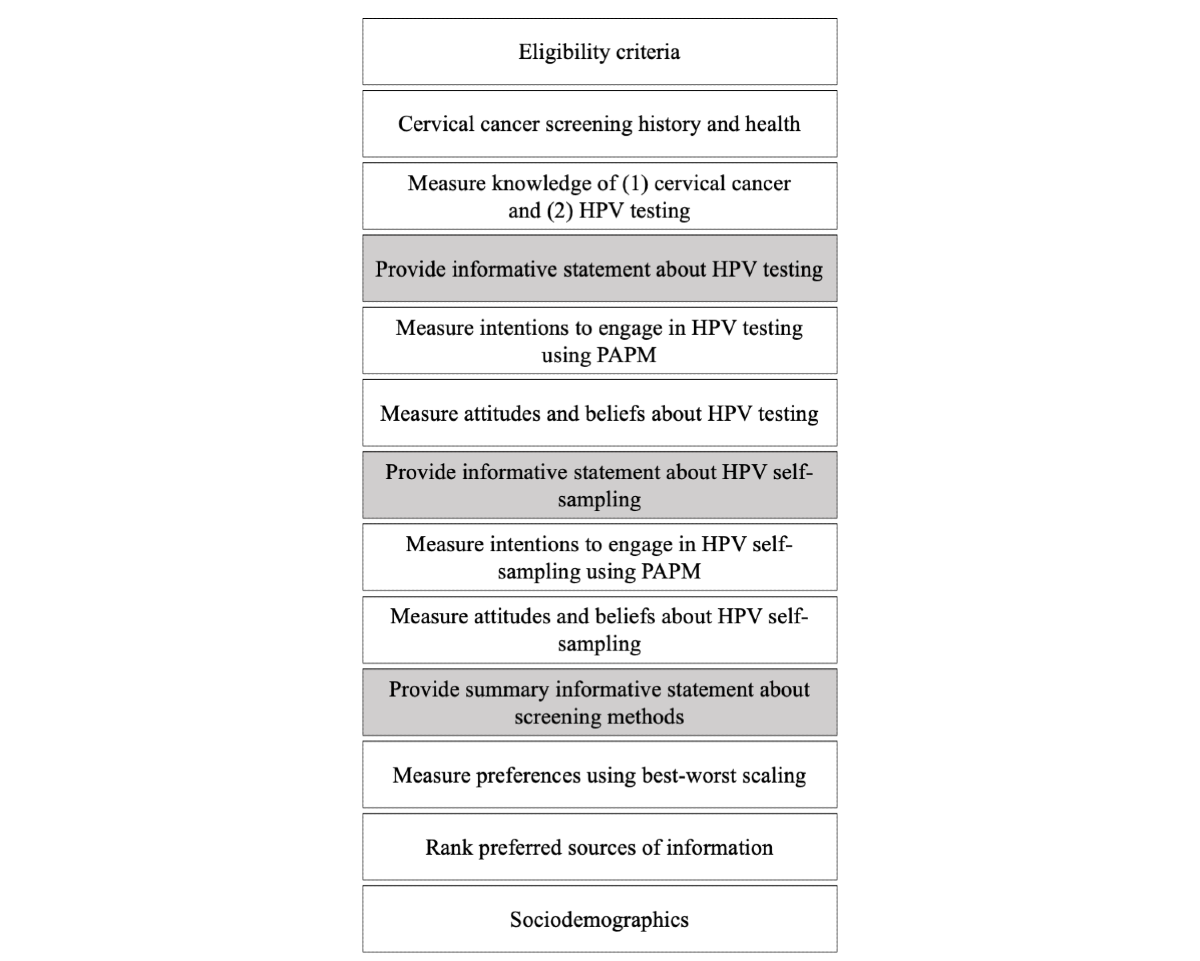
Draft questionnaire structure. HPV: human papillomavirus; PAPM: Precaution Adoption Process Model.

To evaluate potentially relevant items together and guide their selection, a large pool of items (n=781) was created, and a questionnaire structure was drafted (Figure 1) with 4 potential scales: cervical cancer knowledge, HPV testing knowledge, HPV testing attitudes and beliefs, and HPV self-sampling knowledge. This large item pool included items from the reviewed scales and new items generated based on the results of a recent mixed methods synthesis of psychosocial factors affecting HPV test acceptability [24] and a systematic review of emotional responses to testing positive for HPV [25]. Items related to sociodemographics and health behaviors were also included.

**Figure 2:**
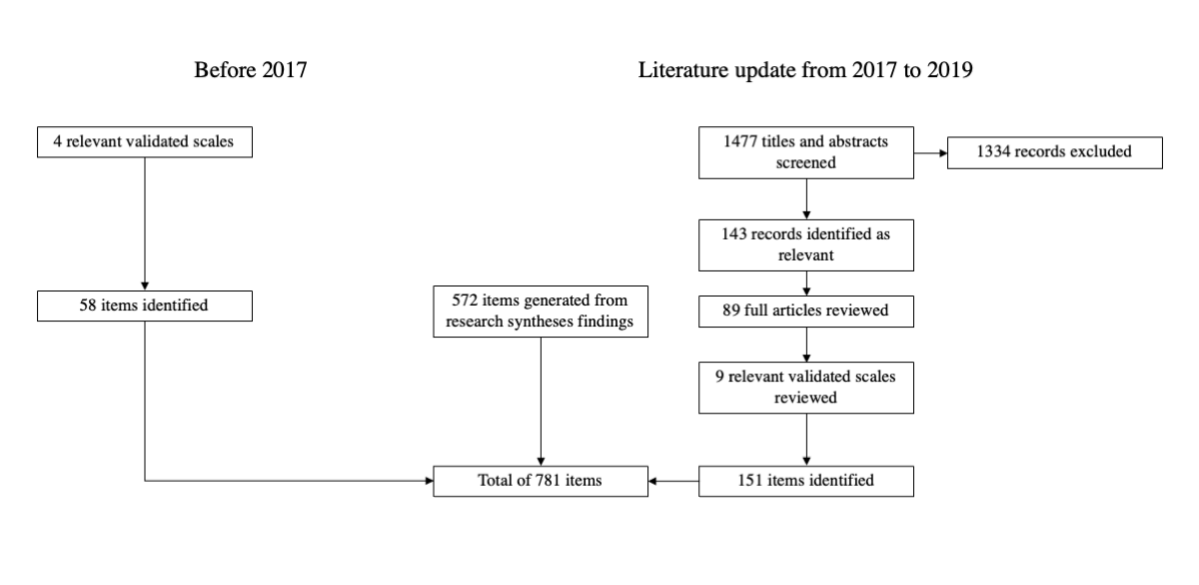
Flowchart of item sources.

All 781 items were compiled into an Excel (Microsoft Corporation) spreadsheet to be reviewed by the research team. A flowchart of the item sources is shown in Figure 2. Each item was reviewed individually over several research team meetings, marked as *retained* or *rejected*, and categorized into an appropriate section of the questionnaire (Figure 1). For instance, the item “The HPV test is safe” was categorized as part of *attitudes and beliefs about HPV testing* instead of *knowledge about HPV testing* because this item captures a belief that women may agree or disagree with, which may impact their intentions to engage in cervical cancer screening with the HPV test. Within each of the questionnaire sections, items were further mapped onto constructs from the HBM (eg, “if the HPV test showed I have HPV, it would be serious” in perceived seriousness) and TPB (eg, “my friends’ opinion about getting the HPV test would be important to me” in social cues) to ensure comprehensive coverage of these frameworks.

At this stage, the focus was on the item’s underlying construct and not its wording. Reasons for rejecting items included the following: (1) duplicate of a factor already retained, (2) not applicable to Canadian context (eg, “There are resources in my community for low and no cost cervical cancer screenings” as Canada has a universal health care system [45]), (3) infrequent and isolated factor (eg, “I worry that tube contents may spoil or spill during transportation to doctor” [63]), and (4) not applicable to our project’s quantitative and survey methodology (eg, open-ended items such as “There are many warning signs and symptoms of cervical cancer. Please name as many as you can think of” [44]).

In total, of 781 items that were reviewed, 137 (17.5%) items were retained. Of these 137 items, 85 items were categorized for scale development: 14 for cervical cancer knowledge, 14 for HPV testing knowledge, 44 for attitudes and beliefs about HPV testing, and 13 for attitudes and beliefs about HPV self-sampling. These 85 items were reviewed and revised separately for clarity, consistency, and grade-8 reading level to account for different language and literacy levels. In addition, certain cervical cancer knowledge and HPV testing knowledge items were revised to achieve a balance of *true* and *false* items (ie, made negative or affirming by adding words such as *not*).

##### Assessing Preferences Using Best-Worst Scaling

To explore women’s preferences for their cervical cancer screening parameters (type of test, screening interval, and age of screening initiation), we designed questionnaire items according to the best-worst scaling (BWS) methodology [64]. Using this methodology, participants’ preferences were examined for different screening intervals (domain A) or various ages of screening initiation (domain B), while also considering multiple screening strategies (ie, Pap test, HPV test, HPV-Pap cotesting, and HPV self-sampling). In domain A, 3 screening intervals were included for assessment according to their applicability to HPV test–based screening implementation: 3 years (the most common interval in Canada for existing cytology-based programs [7]), 5 years (widely recommended for HPV test–based screening [20,65]), and 10 years (implemented for women aged ≥40 years in the Netherlands and considered for wider implementation [66]). In domain B, the following 3 ages of screening initiation were included: 21 years (the most common age of screening initiation in Canada for existing cytology-based programs [7]), 25 years (currently recommended age of screening initiation in several Canadian provinces and widely recommended in countries implementing HPV-based screening in the context of HPV vaccination; eg, the United Kingdom and Australia [7,20]), and 30 years (recommended age of screening initiation with primary HPV-based screening according to the United States Preventive Services Task Force guidelines [18,67]).

##### Informative Statements

Knowledge about HPV in the general population is generally very low [68,69], and knowledge about HPV testing and self-sampling is presumably low among Canadian women because such testing and sampling are not currently part of cervical cancer screening programs. Therefore, we sought to ensure that women had at least basic understanding of HPV testing and HPV self-sampling before examining their attitudes, beliefs, and intentions to screen with these tests. We designed 3 informative statements: one about HPV testing, one about self-sampling using HPV testing, and one comparing cervical cancer screening methods. The development of these statements was inspired by brochures from other countries where HPV-based primary screening is in the process of being implemented or already implemented (eg, the Netherlands, the United Kingdom, and Australia [70-72]). Each informative statement was at most 1 page in length and contained a mix of text, figures, and tables (refer to the example in Multimedia Appendix 1). All informative statements were included after the items that measure participants’ knowledge.

##### Questionnaire Revisions and Translation

A draft version of the questionnaire was circulated to our team of coinvestigators and collaborators (globally based researchers and public health decision makers with expertise in HPV research, cervical cancer screening, epidemiology, oncology, psychometrics, behavior change, and health psychology theory) for review of accuracy of content and feedback. Their suggestions were reviewed and used to refine our questionnaire further (ie, item wording, informative statement content, and questionnaire structure). Then, this version was translated into French by a specialized translation firm, Asiatis, and verified by a native French-speaking member of the research team (GGM).

#### Phase 1B: Questionnaire Validation

Given our objective of developing a comprehensible questionnaire, cognitive interviewing was used as a pretesting method to detect potential sources of errors in the question-answering process [73]. This method permits an assessment of the readability, understanding, and clarity of items to reveal potential differences between the intended meaning of the question from the researcher and the participants’ interpretations [74]. Participants were recruited to provide verbal feedback on their understanding and experience as they completed the questionnaire over Zoom (Zoom Video Communications) with 2 members of the research team being present. Participants read the items as if they were answering the questionnaire and were instructed and prompted to *think-aloud* and provide feedback and suggestions (eg, comment if they did not understand an item or were confused and explain how they reasoned and selected their response) [75]. Each interview lasted between 1 and 2 hours. Our team reviewed the participants’ comments after each interview session and made changes to the questionnaire iteratively to address the issues that arose.

**Figure 3:**
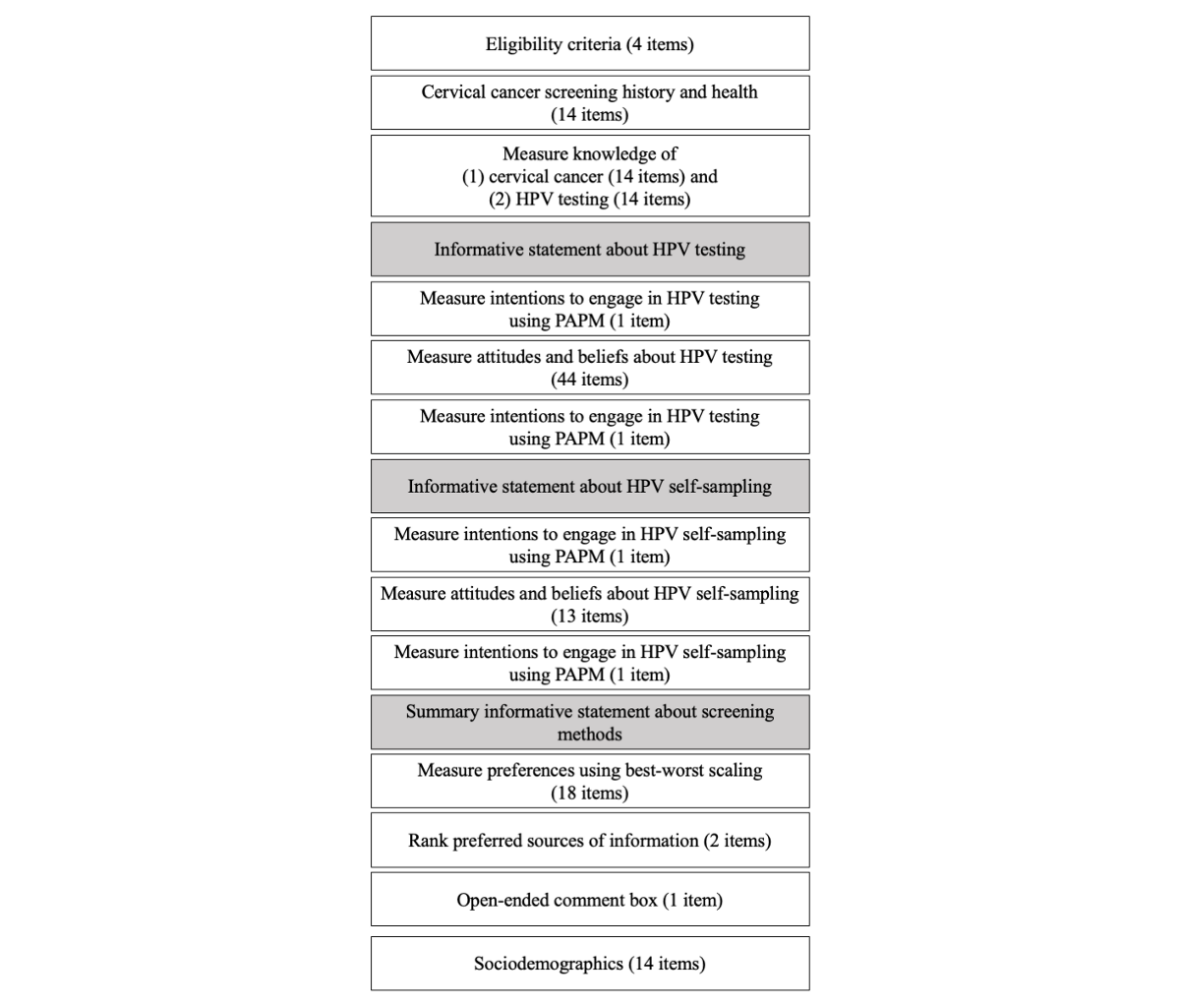
Study 1 questionnaire structure and item count. HPV: human papillomavirus; PAPM: Precaution Adoption Process Model.

The cognitive interviewing phase was completed when no new issues or feedback were raised by the participants. This process required a sample of 7 women, aged 21 to 70 years. Of the 7 interviews, 4 (57%) interviews were conducted in English and 3 (43%) were conducted in French. On the basis of the participants’ feedback, changes were made to the questionnaire to improve the clarity of items, informative statements, instructions, page formatting, and overall questionnaire structure and flow. Refer to Figure 3 for the final structure and item count.

#### Phase 1C: Data Collection and Psychometric Validation

##### Study Design

A cross-sectional web-based survey was administered from October 2021 to November 2021 to a national sample of Canadian women, aged 21 to 70 years, in English and French. Participants completed the questionnaire developed in phases 1A and 1B The survey design and preliminary results are reported according to Checklist for Reporting Results of Internet E-Surveys (CHERRIES; Multimedia Appendix 2) [76]. An electronic consent form describing the study investigators, study goals, procedure, expected survey length, and confidentiality was provided at the start of the survey. Then, the participants were asked whether they consented to taking part in the survey. By clicking on the icon that states they agree to participate, consent was implied, whereas if they declined, the survey was terminated. The information collected during the survey was completely anonymous.

##### Participants

The inclusion criteria were as follows: (1) born female; (2) aged between 21 years, which is the youngest age recommended to begin screening across provincial programs, and 70 years, which is the oldest age; (3) Canadian resident; and (4) intact cervix (eg, not having undergone hysterectomy). The exclusion criterion was having been diagnosed with cervical cancer previously. An oversampling quota was used to ensure that half of the sample was currently underscreened for cervical cancer (ie, >3 years since the previous Pap test or never screened) and the other half was adequately screened (ie, <3 years since the previous Pap test). Census-based quotas were used for primary language and province and territory of residence to reinforce sample representativeness.

##### Overview

The following measures were administered in the order shown in Figure 3. All questions were presented on separate pages of the questionnaire. Participants were not able to go back or review their previous responses, except within each of the psychosocial item sections.

##### Screening History

Participants were asked when they last had a Pap test to assess their screening group: (1) within the past year, (2) within the past 1 to 3 years (adequately screened group), (3) >3 years ago, or (4) never (underscreened group). Then, the participants who reported receiving at least one previous Pap test were asked whether they had ever received an abnormal test result. In addition, given the study’s timing in the fall of 2021, participants who reported receiving a Pap test >3 years ago were asked whether the COVID-19 pandemic had prevented them from receiving a Pap test, as cervical cancer screening was paused in some provinces and territories during parts of the pandemic.

##### Sociodemographics

A total of 4 items used dichotomous response option (yes or no) to measure identification as visible minority [77]; influence of religious or spiritual beliefs on health decisions; living in Canada for ≥10 years; and completion of a trade certificate or diploma, college degree, or university degree. Self-reported ethnic origin was measured with 1 item using the 9 response options recommended by Statistics Canada [78]. We used multiple validated response options to measure gender identity [79]. In addition, household income, employment status, province or territory of residence, and travel time between one’s home and a health care office or clinic were measured.

##### Cervical Cancer–Related Health Behaviors and Risk Factors

Participants answered the following items: self-reported health (from very poor [1] to excellent [6]) [80], use of oral birth control pills for ≥5 years (yes or no), number of children given birth to (0 to ≥10), smoking history (current smoker, smoked in the past, or never smoked), vaccination with at least one dose of an HPV vaccine (yes or no or don’t know), having a family physician (yes or no), height, weight, previous diagnosis of sexually transmitted infection (yes or no), number of lifetime sexual partners, and age of sexual debut.

##### Psychosocial Items for Scale Development

Participants were presented with all items selected as part of phase 1A For the knowledge items, participants responded to each item with *true*, *false*, or *I don’t know*. For attitudes and beliefs items, participants responded to each statement on a 7-point Likert scale ranging from strongly disagree (1) to strongly agree (7). Item presentation was randomized within each section for each participant to mitigate order bias.

##### HPV Testing and Self-sampling Intentions

Using the Precaution Adoption Process Model (PAPM) [81], women selected their current decision-making stage regarding the proposed screening method (HPV testing or self-sampling using the HPV test) from five options: (1) unengaged in the decision to be screened with the HPV test or self-sampling, (2) undecided about whether to be screened with the HPV test or self-sampling, (3) decided not to be screened with the HPV test or self-sampling, (4) decided to be screened with the HPV test or self-sampling, or (5) acted (already screened with the HPV test or self-sampling). A full description of the theoretical background and use of PAPM is provided in the *Study Design* section of study 2.

##### BWS for Screening Preferences

Following the orthogonal main effects design recommended by Aizaki and Fogarty [64] and using the R software packages, *DoE.base* [82] and *support.BWS2* [64], a full set of questions was developed for each of our 2 domains: screening intervals (domain A) and age of screening initiation (domain B). To examine preferences in domains with 4 attributes (ie, screening methods), each of which have 3 attribute levels (ie, screening interval options [domain A] and screening initiation options [domain B]), participants must answer 9 questions that contain the same number of randomly assigned combinations of attribute levels (corresponding to the defined attributes). Therefore, participants answered 18 items; 9 related to cervical cancer screening intervals and 9 related to age of initial screening. Items within each domain were presented randomly for each participant.

##### Preferences for Screening Information and Routine Screening

Using a drag-and-drop interface, participants ranked their preferred source of information from the following options: public health agency website, social media, and health care professional. A similar item was used to assess participants’ preferred type of health care practitioner to administer routine HPV testing among the following: family physician, gynecologist, nurse or nurse practitioner, and physician’s assistant.

##### Recruitment and Data Collection

Recruitment and survey programming were facilitated by Dynata, an international market research firm. Before administering the programmed questionnaire, the research team tested the survey using multiple permutations of response options to ensure its technical functionality and usability and made changes as necessary. Then, Dynata administered the survey to their large panel of Canadian residents who are recruited through *by-invitation-only* method, in which participants’ identities are validated by other partnered businesses to ensure response quality. Eligible participants were invited to complete a survey about *health and wellness* through several platforms, including emails, smartphone app notifications, and Dynata’s website, using a link that is unique to each potential participant. Before beginning the survey, participants’ IP address and unique identifier provided to them by Dynata were used to flag potential duplicate responses. Sample recruitment was conducted over 2 weeks in October 2021 and November 2021. Participants were compensated according to Dynata’s rewards and points system (eg, Amazon and Starbucks). Participants were required to complete all questions before continuing the survey to prevent missing data.

##### Sample Size

We calculated the minimum sample size for conducting factor analyses using the criteria recommended by Mundfrom et al [83], which include the expected ratio of variables to factors, level of communality, and agreement between the sample and population solutions (K). On the basis of ratio of variables to factors of 4 (for the 13 items reflecting attitudes and beliefs for sampling), low level of communality (ie, range 0.2-0.4), and excellent agreement between the sample and population solutions (ie, K=0.98), the minimum sample required was 500 (rounded up from 450). As our analytic plan includes conducting EFA and confirmatory factor analysis (CFA) on separate samples and considering that oversampling is needed to account for approximately 18% invalid and inattentive responses [84], the sample needed is 1220 observations, that is, (2 × 500 × 100) / 82 = 1219.

##### Data Cleaning

Direct and statistical data cleaning methods were used stepwise to identify potentially *inattentive* or *unmotivated* respondents in our final data set of completed questionnaire responses. In total, 2 attention check items were used in the survey: one within the items related to HPV test attitudes and beliefs scale and one in the items related to HPV self-sampling attitudes and beliefs. Following the instructed response design [84], each of these items asked participants to select a specific response choice from a Likert scale to verify attention (eg, “please select ‘strongly agree,’ for this question only”). Participants who responded correctly to at least one of these items were retained. Next, participants who *straight-lined* (ie, answered all items using the same response) were identified by calculating the variance in response for all the HPV testing attitudes and beliefs items. Participants who did not exhibit any variance in their responses across these items were excluded. Finally, among the remaining participants, those in the longest 2.5% and shortest 2.5% of the survey response times were excluded.

##### Data Analysis—Objective 1: Scale Validation

To evaluate dimensionality, we will conduct EFA and CFA separately for cervical cancer knowledge, HPV testing knowledge, and HPV testing and self-sampling attitudes and beliefs items. The final data set will be split randomly into 2 equal samples, and one sample will be used to conduct EFA and the other sample will be used to validate the factor structure using CFA. To select the optimal number of factors, we will use the parallel analysis approach and the syntax developed by O’Connor [85]. For items within each factor, we will use item response theory modeling; for binary data (knowledge items), we will use the 2-parameter logistic regression model that accounts for item difficulty and discriminant capacity; and for ordinal data (attitudes and beliefs items), we will use the graded response model that accounts for discriminant capacity and probability of selecting a certain Likert scale score (eg, strongly agree). Concurrently, we will examine how well each item measures the latent trait by plotting the item information against the latent construct ability. We aim to retain items that cover a wide range of difficulty and have high discriminant capacity and information value. To estimate the CFA model fit, we will use the fit indices and cutoff criteria recommended by Hooper et al [86]: (1) relative (normed) chi-square test (*χ*^2^/_df_=2 to 5) by Wheaton et al [87], (2) standardized root mean square residual (<0.8), (3) root mean square error approximation (<0.06), (4) comparative fit index (≥0.95), and (5) Tucker-Lewis index (≥0.95). To evaluate internal consistency for items loading onto each factor, Cronbach *α* will be calculated.

###### BWS Preferences

To analyze BWS preference data, we will use the counting and modeling approaches described by Aizaki and Fogarty [64]. Consistent with the counting approach, for domain A (screening interval) and domain B (age of initiation), we will calculate the best-minus-worst score (higher scores reflect higher preferences) for each attribute (eg, HPV test) and attribute level (eg, 3 years [for screening interval] and 25 years [for age of initiation]). For the modeling approach, we will use conditional logistic regression to model preferences as a function of the sum of the values of attributes and attribute levels [64]. For attributes and attribute levels, we will estimate odds ratios and 95% CIs.

### Results

#### Overview

In total, 83.49% (1027/1230) valid responses were retained for analysis following data cleaning. Data analysis is ongoing. Results of study 1, including findings from the BWS analyses and the development of cervical cancer knowledge, HPV testing knowledge, HPV testing attitudes and beliefs, and HPV self-sampling attitudes and beliefs scales are expected to be published in the summer and fall of 2022.

#### Objective 2: Questionnaire Feasibility

**Figure 4:**
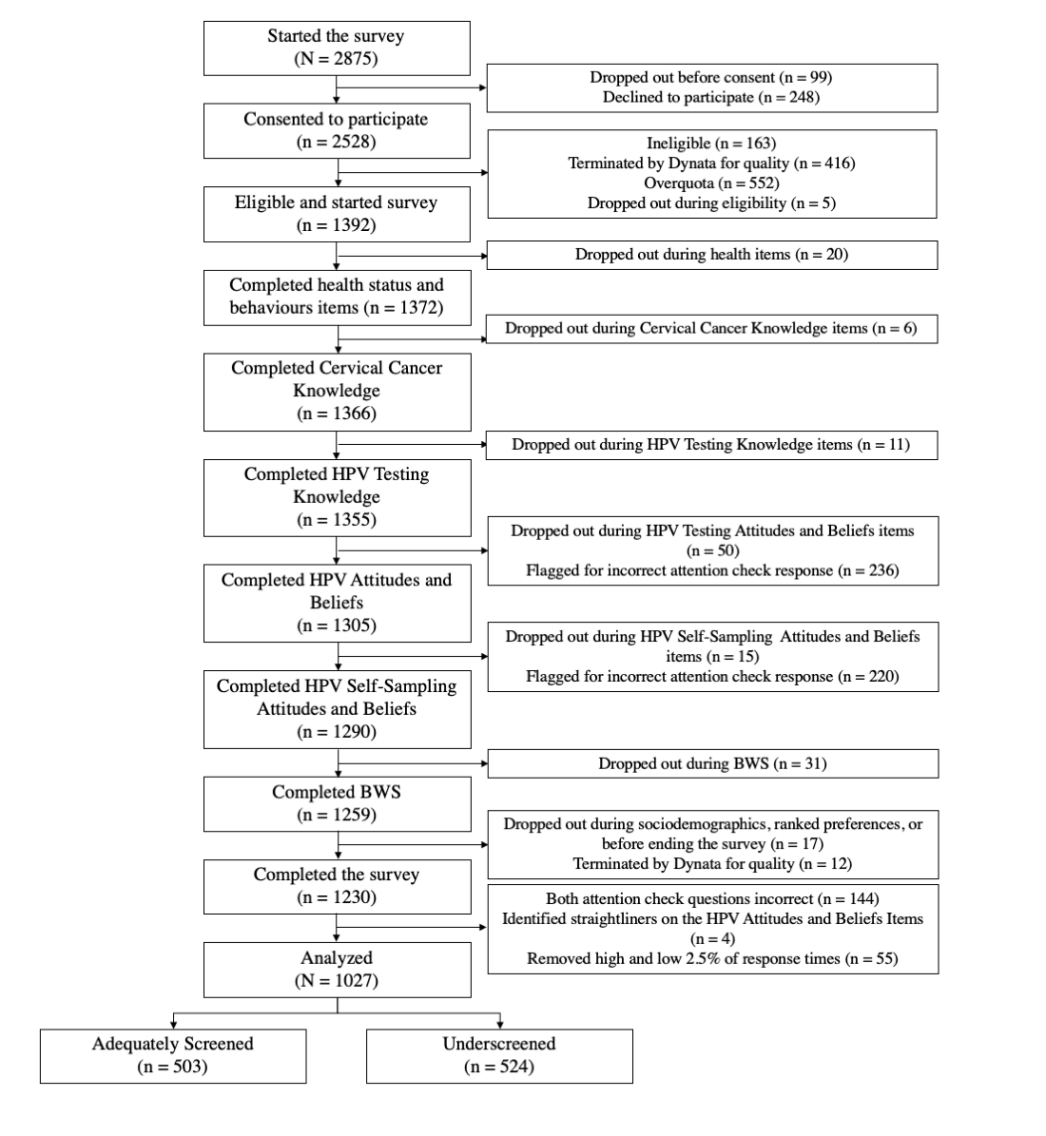
Study 1—participant flow diagram. BWS: best-worst scaling; HPV: human papillomavirus.

Preliminary results suggest high level of feasibility. Through the recruitment methods of Dynata, data collection was completed within 2 weeks. As detailed in the *Data Cleaning* section, overall, data were of good quality, with the exclusion of 16.5% (203/1230) of complete responses owing to poor quality, being consistent with previous research in our laboratory and expectations for survey research [39,84,88]. The mean response time (after data cleaning) was 30.4 (SD 20.5) minutes, consistent with other surveys conducted by our research team [89]. It is possible that some participants left the survey and returned to it later, which could explain the slightly higher response time than our estimated 25 minutes. Given that the scales in study 2 will be made more concise through psychometric analyses applied to responses from this study, we expect the survey in study 2 to be shorter in length. Of the 1392 participants who were eligible and began the survey, 1230 (88.36%) participants completed the survey, with an attrition rate of 11.64% (162/1392) (refer to Figure 4 for a diagram detailing participant attrition). Attrition primarily occurred in the HPV attitudes and beliefs section (50/162, 30.86%), which is the longest section of the survey, with 44 items. We expect that the validation and shortening of this scale for study 2 will help to address this issue.

## Study 2: Expanded Population-Based Survey of Canadian Women

### Methods

#### Study 2: Ethics Approval

Study 2 received approval from the research ethics board of the Centre intégré universitaire de santé et de services sociaux West-Central Montreal (project ID 2022-2960) in July 2021. Informed consent will follow the same procedure as that in study 1.

#### Study Design

A cross-sectional web-based survey will be administered in summer of 2022 to a nationally representative sample of Canadian women, aged 21 to 70 years, in English and French. The study population and inclusion criteria will be the same as those in study 1. Oversampling will be used to ensure that half of the sample is adequately screened and the other half is underscreened. Additional quotas will be used for the following factors: age, household income, and rural or urban residence (considering low cervical screening rates in rural Canada [7]) based on census data from Statistics Canada. Data collection will be conducted by Dynata, following the same procedure as that in study 2.

The questionnaire will follow the same structure and include the same sections as the survey detailed in study 1. Of the knowledge, attitudes, and beliefs items used for scale development in study 1, only those items retained after extensive psychometric analyses will be used in the study 2 questionnaire as part of the resulting shorter, validated scales. In addition, 2 previously validated measures, the extended HPV General Knowledge and HPV Vaccine Knowledge scales, will be added [69], given the relevance of HPV and HPV vaccination in cervical cancer. The measurement of the outcome variables (intentions to screen using the HPV test and self-sampling) is informed by PAPM [81], a categorical, stage-based model of health decision-making. As a binary, *yes or no* outcome limits accuracy, using a multistage model provides a more precise, nuanced understanding of women’s decision-making process that acknowledges the unique barriers associated with movement between each stage toward engaging with the behavior [90].

#### Sample Size

We calculated the sample size based on the estimation that approximately 30% of women will be in each of the PAPM adoption stages, *unengaged*, *undecided*, and *decided to*, and 10% will be in the *decided not* stage*.* As multinomial logistic regression can be considered as a series of binary logistic regressions, we based our calculations on the work of Peduzzi et al [91], which recommends a minimum of 10 observations per variable and the formula, N=10k/p, where N is the minimum number of observations needed, k is the number of predictor variables, and p is the smallest proportion in the binary model, that is, N = (10 × 15) / 0.25 = 600. Therefore, the minimum sample needed to reach sufficient power is 1500 because 40% of the sample (eg, unengaged+decided not) must represent 600 observations. Given that our objective is to estimate the association between psychosocial factors and intentions of HPV testing in both underscreened women and adequately screened women, we need 3000 (ie, 1500 in each group) valid responses to perform the analyses. Considering that an oversampling of maximum of 18% is required to account for careless responses, the approximate total number of survey responses needed is 3650.

#### Data Analysis

Corresponding to objective 1, we will conduct univariate analyses (means and proportions) for scale items (eg, knowledge and attitudes) and bivariate analyses (2-tailed *t* tests and chi-square tests) to estimate the differences between adequately screened women and underscreened women. We will calculate the effect size using Cohen *h* and Cohen *d* for proportions and continuous variables, respectively. Corresponding to objective 2, we will estimate the associations between psychosocial factors and intentions of HPV testing and self-sampling using multinomial logistic regression and model the log odds of PAPM stages (dependent variable) as a linear combination of the independent variables (eg, HPV testing knowledge and BWS preference scores). We will report odds ratios and 95% CIs of being in a PAPM stage versus the reference category (ie, unengaged) for each independent variable. Initially, we will conduct bivariate analyses to estimate the association between the outcome and independent variables. Then, independent variables showing significant associations (*P*<.10) will be entered simultaneously in the final model. We selected the following indices to report model fit: (1) Cox-Snell *R*^2^, (2) Cragg-Uhler *R*^2^, and (3) McFadden *R*^2^ [39,92]. We will use the Hausman-McFadden test to evaluate the final model for independence of irrelevant alternatives, which postulates that a person’s choice (ie, PAPM stage) is unchanged by other available choices (ie, fewer PAPM stages) [93]. Analyses will be conducted separately for underscreened women and adequately screened women. Analyses will be conducted using SPSS (IBM Corporation), R software (R Foundation for Statistical Computing), and STATA (StataCorp).

### Results

As of May 2022, data collection has not begun for study 2. Data collection is expected to begin in June 2022 or July 2022, and results are expected to be published in late 2022 and early 2023.

## Discussion

### Principal Findings

This study will use 2 complementary cross-sectional web-based surveys of Canadian women to develop psychometrically tested scales measuring cervical cancer knowledge, HPV testing knowledge, HPV testing attitudes and beliefs, and HPV self-sampling attitudes and beliefs and, then, include these scales in a broad survey to investigate the psychosocial correlates of women’s intentions to engage in HPV-based cervical cancer screening.

Scales developed in study 1 will be informed by measures in the extant literature and further enhanced by the generation of new items based on systematic evaluation of themes in the psychosocial literature on HPV testing. Using the TPB and HBM to guide item selection will further ensure that the scales provide meaningful insights into the factors affecting screening behaviors. Application of advanced psychometric methods, including item response theory, which facilitates the development of parsimonious measures [94], will help to ensure that the scales are comprehensive, while being brief and easy to administer. We expect the cervical cancer knowledge scale to expand on the psychometric methods applied for similar measures such as the Cervical Cancer Awareness Measure developed by Simon et al [44] and the HPV testing knowledge scale to show increased reliability compared with the existing subscale developed by Waller et al [48]. The HPV testing and self-sampling attitudes and beliefs scales will expand on existing measures by using advanced psychometrics in an up-to-date and representative sample. Accordingly, we expect our scales to represent a new measurement standard for investigating the attitudes, beliefs, and knowledge associated with HPV-based screening. In addition, the use of BWS to estimate preferences for cervical cancer screening will enable evaluation of women’s perceptions of trade-offs between different testing methods, and importantly, screening intervals and ages of initial screening. These changes have been barriers in other countries where HPV-based screening has been implemented [22,52].

Our expanded population-based survey (study 2) will provide comprehensive data to inform and support the development of Canadian HPV-based cervical cancer screening programs. Through objective 1, the specific evaluation of differences in knowledge, attitudes, and beliefs about HPV testing among women who are underscreened and those who are adequately screened will enable the development of targeted interventions to improve knowledge, address negative attitudes and beliefs, and reassure women about upcoming changes. In objective 2, analyzing the sociodemographic and psychosocial factors associated with screening intention stages will provide insight into developing interventions that consider the multiplicity of barriers to screening. Our investigation will offer a valuable response to calls to action to examine inequalities in cervical cancer screening in the interest of cervical cancer elimination [8]. Furthermore, by comparing women’s knowledge, attitudes, beliefs, and preferences toward HPV-based screening with the proposed guidelines, our findings will help to inform screening recommendations and ensure successful transition from cytology to HPV-based screening in Canada.

### Knowledge Dissemination Plan

To reach both research and health professional audiences, the study findings will be published in open-access, peer-reviewed scientific journals. Presentations will be made at national and international scientific meetings (eg, Canadian Association of Psychosocial Oncology, International Papillomavirus Society conference, and International Psycho-Oncology Society) and in webinars (eg, Consortium for Infectious Disease Control and International Papillomavirus Society). To reach policy makers, we will share a final research report with the Public Health Agency of Canada, CPAC, Canadian Task Force on Preventive Health Care, and provincial and territorial cancer screening program advisory boards. We will also share our results with nonprofit organizations such as the Canadian Cancer Society, the Society of Obstetricians and Gynaecologists of Canada, the College of Family Physicians of Canada, and HPV Global Action who have expressed strong interest in disseminating our results. We will produce content (eg, infographics) and engage with Canadian women on social media (Twitter, Facebook, etc). Research summaries will be drafted for dissemination to national media outlets to inform women about this public policy change and encourage discussions about HPV-based cervical cancer screening.

### Strengths and Limitations

A major strength of this study is the rigorous and comprehensive process to develop psychometrically validated scales informed by theoretical frameworks. In addition, examination of the feasibility of the developed questionnaire through study 1 will ensure that the survey used in study 2 will be easy to understand and relevant to Canadian women. BWS presents an innovative approach to assess preferences that controls for biases observed in typical multiple-choice or ranking assessments of preferences. The use of PAPM to examine intentions toward HPV testing and self-sampling will provide a theoretically informed and nuanced understanding of women’s decision-making compared with other studies using continuous or binary measures. Using a market research polling firm will enable time-efficient and cost-efficient recruitment and data collection. Furthermore, the web-based survey methodology will prevent the issue of missing data. Quota-based sampling will allow us to recruit a nationally representative sample based on recent census data. Attention check items and data cleaning techniques will allow us to identify and exclude unmotivated or *careless* responders, ensuring that high-quality data will be collected and analyzed.

The study design has some limitations. Relying on respondents’ self-report for their screening history of having had a Pap test in the past 3 years or not is subject to recall bias. Our anonymous web-based survey design prevents us from verifying it against health records data. To minimize this limitation, women will be provided with a reminder informative statement explaining what a Pap test is and how it is performed before asking them about their screening history. Given that the COVID-19 pandemic has prevented and continues to prevent women from engaging in cervical cancer screening, this may affect women’s report of their screening history and change the composition of women in our *underscreened* and *adequately screened* categories. It is not clear how this will affect our data, as the effect of the COVID-19 pandemic on screening access in Canada is not well understood in most provinces and territories [95]. To address this issue, we will include an item asking those participants who report being underscreened whether the COVID-19 pandemic had prevented them from receiving screening, and sensitivity analyses will be performed to examine the pandemic’s impact on screening. In addition, the lack of investigation at 2 different time points precludes any investigation of how intentions for HPV testing and self-sampling may relate to uptake and acceptability as these testing methods are introduced. Longitudinal examinations of acceptability and intentions toward HPV-based screening will be needed as implementation occurs in Canada. Unfortunately, our study design and recruitment strategy may preclude specific analyses in gender and sexual minority groups, considering the low case counts observed in population-wide web surveys (eg, 0.4% gender-diverse individuals in study 1). Recognizing the unique barriers faced by these groups in screening for cervical cancer, future research should specifically investigate the perceptions of gender and sexual minority Canadians toward HPV testing and self-sampling implementation [3,14,96]. Similarly, comprehensive understanding of cervical cancer screening and HPV in the First Nations, Inuit, and Métis populations is critical [5,34]. Our study could highlight certain issues in these populations (considering 2.9% representation in study 1), but unique participatory action initiatives involving relevant nongovernmental organizations and community advocacy groups are needed to fully understand their views and address their concerns.

### Conclusions

Understanding the psychosocial factors that might affect women’s perceptions of and intentions to engage in HPV-based screening will be critical as Canada plans to implement changes to cervical screening programs and guidelines. Through this multistep study, we will develop several validated scales to facilitate population-based investigations of these factors by other researchers. The use of these scales to investigate a representative sample of Canadian women’s perceptions of HPV-based screening will provide directly applicable knowledge to public health and health care professionals.

## Appendix

Multimedia Appendix 1 Example informative statement.

Multimedia Appendix 2 Checklist for Reporting Results of Internet E-Surveys (CHERRIES) checklist.

Multimedia Appendix 3 Canadian Institutes of Health Research—external peer review reports.

Multimedia Appendix 4 Study 1—detailed questionnaire outline with sample items.

## Abbreviations

BWS: best-worst scaling
CFA: confirmatory factor analysis
CHERRIES: Checklist for Reporting Results of Internet E-Surveys
CIHR: Canadian Institutes of Health Research
CPAC: Canadian Partnership Against Cancer
EFA: exploratory factor analysis
HBM: Health Belief Model
HPV: human papillomavirus
PAPM: Precaution Adoption Process Model
TPB: Theory of Planned Behavior

## Data Availability

The data sets that will be generated through this study will not be published in a publicly available repository in accordance with the ethics proposal approved by the overseeing research ethics board. They will be available from the senior author (ZR) on reasonable request and upon agreement of confidentiality and data use policies provisioned by the primary institution. The full questionnaire used in the study will be available upon request to the corresponding author, and a detailed questionnaire outline for study 1, including several sample items for each section, is available in Multimedia Appendix 4.
