# Supplementary figures and images for: Ensuring a Successful Transition From Cytology to Human Papillomavirus–Based Primary Cervical Cancer Screening in Canada by Investigating the Psychosocial Correlates of Women’s Intentions: Protocol for an Observational Study
Source: JMIR Res Protoc. 2022 Jun 16;11(6):e38917. doi: 10.2196/38917 (PMC9247817; doi:10.2196/38917)

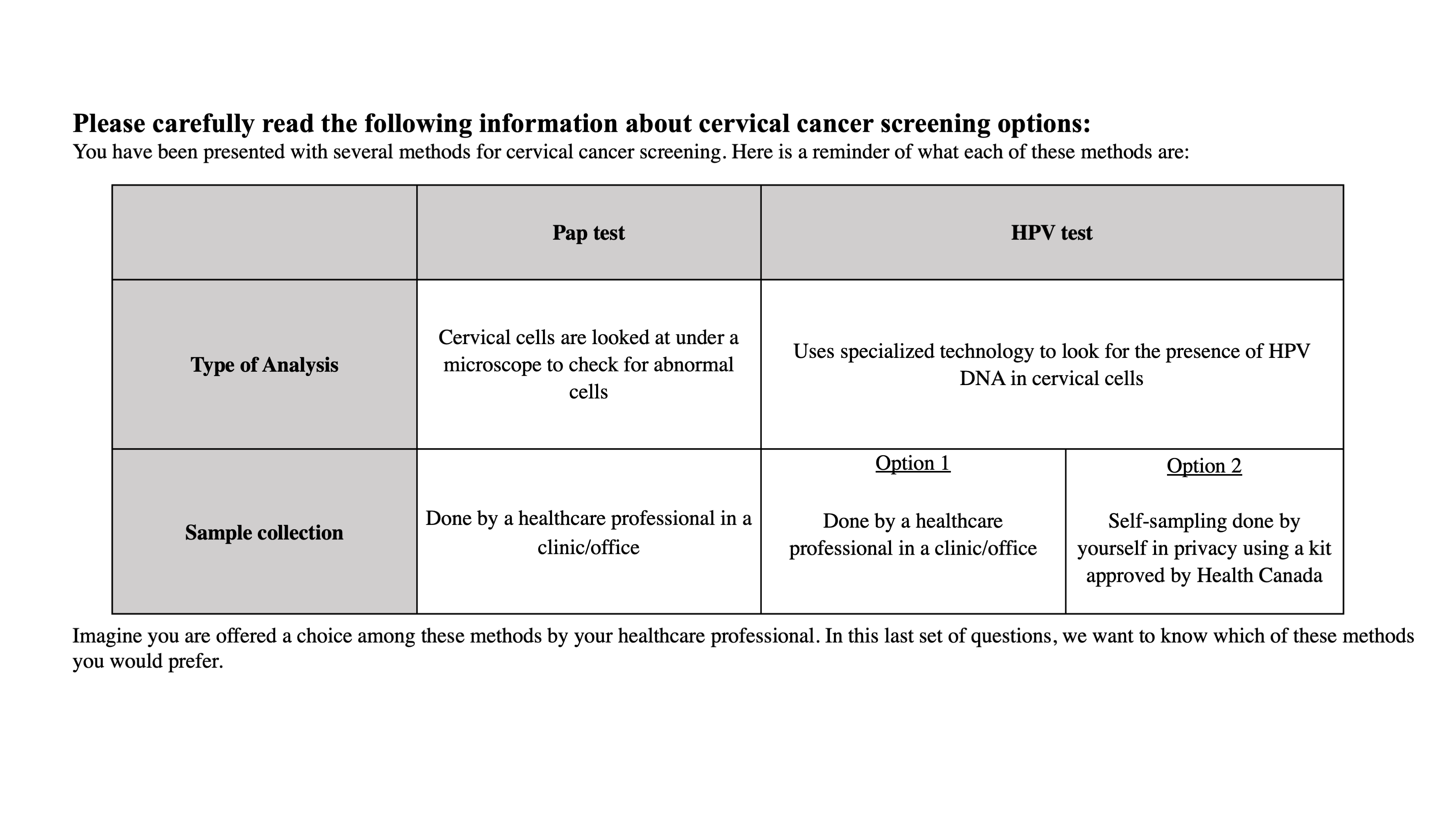

Supplement: Multimedia Appendix 1 [file resprot_v11i6e38917_app1.png]
