# Supplementary material for: Ensuring a Successful Transition From Cytology to Human Papillomavirus–Based Primary Cervical Cancer Screening in Canada by Investigating the Psychosocial Correlates of Women’s Intentions: Protocol for an Observational Study
Source: JMIR Res Protoc. 2022 Jun 16;11(6):e38917. doi: 10.2196/38917 (PMC9247817; doi:10.2196/38917)
Supplement: Multimedia Appendix 3 [file resprot_v11i6e38917_app3.pdf]

**Review Type / Type d'évaluation:**	Reviewer 1 / Évaluateur 1
**Name of Applicant / Nom du chercheur:**	Rosberger, Zeev
**Application No. / Numéro de demande:**	420029
**Agency / Agence:**	CIHR/IRSC
**Competition / Concours:**	Project Grant/Subvention Projet
**Committee / Comité:**	Public, Community & Population Health/Santé publique, santé communautaire et santé des populations
**Title / Titre:**	Ensuring a Successful Transition from Pap to HPV DNA Testing in Primary Cervical Cancer Screening: Exploring and Listening to Canadian Women's Needs is Critical for Effective Public Policy Change

Adjudication Criteria/Critères de sélection

Significance and Impact of the Research/Importance et impact de la recherche: 4.5

Approaches and Methods/Approches et méthodes: 4.3

Expertise, Experience and Resources/Expertise, expérience et ressources: 4.3

Top/Bottom Selection/Groupe supérieur/inférieur

- (●) **Top/Groupe supérieur**
- ( ) **Bottom/Groupe inférieur**

**Review Type / Type d'évaluation:**	Reviewer 1 / Évaluateur 1
**Name of Applicant / Nom du chercheur:**	Rosberger, Zeev
**Application No. / Numéro de demande:**	420029
**Agency / Agence:**	CIHR/IRSC
**Competition / Concours:**	Project Grant/Subvention Projet
**Committee / Comité:**	Public, Community & Population Health/Santé publique, santé communautaire et santé des populations
**Title / Titre:**	Ensuring a Successful Transition from Pap to HPV DNA Testing in Primary Cervical Cancer Screening: Exploring and Listening to Canadian Women's Needs is Critical for Effective Public Policy Change

Summary of Application/Résumé de la demande:

This research proposes to assess Canadian women’s understanding of HPV DNA testing as preventive of cervical cancer. The research is timely given that many provinces plan to implement screening programs using this method, but it is unknown how Canadian women will respond. A similar program in Australia was delayed because of a backlash due to misunderstanding of the process. The measures are based on TPB and HBM and women will be categorized into five intention stages according to stages in the Precaution Adoption Process Model. This is more subtle an outcome than a binary yes/no intention to be screened. Their objectives are to 1) estimate knowledge differences between underscreened and adequately screened women (based on PAP); 2) to estimate differences in attitudes and beliefs about the HPV test between underscreened and adequately screened women; 3) to assess which psychosocial factors may be related to intentions to be screened with the HPV test; and 4) to assess which psychosocial factors may be related to intentions to use self-sampling for HPV testing.

**Review Type / Type d'évaluation:**	Reviewer 1 / Évaluateur 1
**Name of Applicant / Nom du chercheur:**	Rosberger, Zeev
**Application No. / Numéro de demande:**	420029
**Agency / Agence:**	CIHR/IRSC
**Competition / Concours:**	Project Grant/Subvention Projet
**Committee / Comité:**	Public, Community & Population Health/Santé publique, santé communautaire et santé des populations
**Title / Titre:**	Ensuring a Successful Transition from Pap to HPV DNA Testing in Primary Cervical Cancer Screening: Exploring and Listening to Canadian Women's Needs is Critical for Effective Public Policy Change

Strengths and Weaknesses/Forces et faiblesses:

The researchers make a very strong case for the timeliness of this research. The change in practice will occur but uptake problems could be prevented if the beliefs of women are taken into account. They also argue that there is no Canadian data available (based on a systematic review they conducted).

The theoretical basis for the study is well thought out and the measures are appropriately based on theory.

The research is based on previous work by the team that includes a systematic review outlining some of the issues in the area. The team itself is strong with relevant expertise and the inclusion of knowledge users.

The researchers argue that women who do not have English or French as their first language are less likely to be screened, but this is not addressed in the research design. They include a comment in the limitations about this and that the results will inform research with marginalized groups, including Indigenous groups. If the research is needed now, how will these groups be helped? The researchers made a strong case for the need for research with underrepresented groups at the start, and for the timeliness of the research, but it appears that these issues will not be addressed by the currently proposed research. This is the fourth time this research was submitted. In reading the application (before reading the response to reviewers) I had the concern that vulnerable groups were not included. Unfortunately, the response to reviewers does not increase my confidence. How will the market survey company ensure women who do not speak English or French will complete a survey only offered in those two languages? I think it is best to just acknowledge this is a limitation.

I'm not sure what is meant by “intelligent programming of the survey” – is this from the market survey company?

The researchers will ask women the date of their last gynecological exam because they are concerned they will not remember when their last pap test was, but will women be able to remember their last gynecological exam any better than when their last pap was?

I see this research as an important first step, but it is unclear how the results will actually be used to mitigate potential concerns among Canadian women. The researchers propose a one day workshop to present findings to policy makers and media, but how those people disseminate the findings to the public is not within the scope of this research.

I suggest the informative statements be simplified, particularly if they want to get adequate data from lower SES or immigrant groups.

**Review Type / Type d'évaluation:**	Reviewer 1 / Évaluateur 1
**Name of Applicant / Nom du chercheur:**	Rosberger, Zeev
**Application No. / Numéro de demande:**	420029
**Agency / Agence:**	CIHR/IRSC
**Competition / Concours:**	Project Grant/Subvention Projet
**Committee / Comité:**	Public, Community & Population Health/Santé publique, santé communautaire et santé des populations
**Title / Titre:**	Ensuring a Successful Transition from Pap to HPV DNA Testing in Primary Cervical Cancer Screening: Exploring and Listening to Canadian Women's Needs is Critical for Effective Public Policy Change

Budget Recommendation/Recommandation budgétaire:

No concerns

**Review Type / Type d'évaluation:**	Reviewer 1 / Évaluateur 1
**Name of Applicant / Nom du chercheur:**	Rosberger, Zeev
**Application No. / Numéro de demande:**	420029
**Agency / Agence:**	CIHR/IRSC
**Competition / Concours:**	Project Grant/Subvention Projet
**Committee / Comité:**	Public, Community & Population Health/Santé publique, santé communautaire et santé des populations
**Title / Titre:**	Ensuring a Successful Transition from Pap to HPV DNA Testing in Primary Cervical Cancer Screening: Exploring and Listening to Canadian Women's Needs is Critical for Effective Public Policy Change

Please indicate your appraisal of the integration of sex as a biological variable as a strength, weakness, or not applicable to the proposal./Prière de sélectionner une option pour donner votre évaluation de l'intégration du sexe comme variable biologique en tant que point fort ou point faible de la proposition, ou en tant qu'élément non applicable à la proposition.

- (●) **Strength/Point fort**
- ( ) **Weakness/Point faible**
- ( ) **Not applicable/Non applicable**

Please indicate your appraisal of the integration of gender as a socio-cultural determinant of health as a strength, weakness, or not applicable to the proposal./Prière de sélectionner une option pour donner votre évaluation de l'intégration du genre comme déterminant socioculturel de la santé en tant que point fort ou point faible de la proposition, ou en tant qu'élément non applicable à la proposition.

- (●) **Strength/Point fort**
- ( ) **Weakness/Point faible**
- ( ) **Not applicable/Non applicable**

**Review Type / Type d'évaluation:**	Reviewer 1 / Évaluateur 1
**Name of Applicant / Nom du chercheur:**	Rosberger, Zeev
**Application No. / Numéro de demande:**	420029
**Agency / Agence:**	CIHR/IRSC
**Competition / Concours:**	Project Grant/Subvention Projet
**Committee / Comité:**	Public, Community & Population Health/Santé publique, santé communautaire et santé des populations
**Title / Titre:**	Ensuring a Successful Transition from Pap to HPV DNA Testing in Primary Cervical Cancer Screening: Exploring and Listening to Canadian Women's Needs is Critical for Effective Public Policy Change

Sex and/or Gender Considerations/Notions de sexe et/ou de genre:

Inherent to the design.

**Review Type / Type d'évaluation:**	Reviewer 2 / Évaluateur 2
**Name of Applicant / Nom du chercheur:**	Rosberger, Zeev
**Application No. / Numéro de demande:**	420029
**Agency / Agence:**	CIHR/IRSC
**Competition / Concours:**	Project Grant/Subvention Projet
**Committee / Comité:**	Public, Community & Population Health/Santé publique, santé communautaire et santé des populations
**Title / Titre:**	Ensuring a Successful Transition from Pap to HPV DNA Testing in Primary Cervical Cancer Screening: Exploring and Listening to Canadian Women's Needs is Critical for Effective Public Policy Change

Adjudication Criteria/Critères de sélection

Significance and Impact of the Research/Importance et impact de la recherche: 4.5

Approaches and Methods/Approches et méthodes: 4.0

Expertise, Experience and Resources/Expertise, expérience et ressources: 4.2

Top/Bottom Selection/Groupe supérieur/inférieur

- (●) **Top/Groupe supérieur**
- ( ) **Bottom/Groupe inférieur**

**Review Type / Type d'évaluation:**	Reviewer 2 / Évaluateur 2
**Name of Applicant / Nom du chercheur:**	Rosberger, Zeev
**Application No. / Numéro de demande:**	420029
**Agency / Agence:**	CIHR/IRSC
**Competition / Concours:**	Project Grant/Subvention Projet
**Committee / Comité:**	Public, Community & Population Health/Santé publique, santé communautaire et santé des populations
**Title / Titre:**	Ensuring a Successful Transition from Pap to HPV DNA Testing in Primary Cervical Cancer Screening: Exploring and Listening to Canadian Women's Needs is Critical for Effective Public Policy Change

Summary of Application/Résumé de la demande:

Rosberger and colleagues note that the cervical cancer prevention landscape is shifting in Canada. Current guidelines focus on identification of cervical neoplasia using Pap methods at relatively short intervals; however, there is increasing acceptance by expert guidance bodies of screening practices with wider intervals, later age of onset, and (increasingly) HPV testing (with self-sampling). As they note, such major changes in practice must be informed by information on knowledge, beliefs, preferences and practices of Canadian women, and such knowledge does not currently exist in Canada.

They propose to perform a web-based survey in partnership with Leger, which will compare knowledge, attitudes and practices around cervical cancer screening in adequately screened and underscreened women. There is a particular emphasis on attitudes and knowledge related to HPV testing, which is key, given impending changes in cervical cancer screening.

**Review Type / Type d'évaluation:**	Reviewer 2 / Évaluateur 2
**Name of Applicant / Nom du chercheur:**	Rosberger, Zeev
**Application No. / Numéro de demande:**	420029
**Agency / Agence:**	CIHR/IRSC
**Competition / Concours:**	Project Grant/Subvention Projet
**Committee / Comité:**	Public, Community & Population Health/Santé publique, santé communautaire et santé des populations
**Title / Titre:**	Ensuring a Successful Transition from Pap to HPV DNA Testing in Primary Cervical Cancer Screening: Exploring and Listening to Canadian Women's Needs is Critical for Effective Public Policy Change

Strengths and Weaknesses/Forces et faiblesses:

This is a strong proposal which has been extensively and repeatedly revised to meet the concerns of reviewers. The approach is reasonable, and the work is important.

The background is well written; the team are expert in areas of cervical cancer prevention and survey research, including work related to cervical cancer prevention. The work is very timely.

Measurements are to be made using validated available instruments and approaches. I do not see mention of survey weights in the proposal itself but provision of appropriate weights is noted in the materials provided by Leger.

There are sensible approaches to oversampling, and to dealing with careless responses and non-completion. The sample size description is reassuring. Again, as above, these investigators have knowledge and experience in this area.

The obvious criticism here is that women who are disenfranchised enough to be non-participants in existing cervical cancer screening programs are likely to be underrepresented among women with internet access. This is a limitation but I don't have any suggestion as to how this could be reasonably overcome, and the work remains of substantial importance even with this limitation.

**Review Type / Type d'évaluation:**	Reviewer 2 / Évaluateur 2
**Name of Applicant / Nom du chercheur:**	Rosberger, Zeev
**Application No. / Numéro de demande:**	420029
**Agency / Agence:**	CIHR/IRSC
**Competition / Concours:**	Project Grant/Subvention Projet
**Committee / Comité:**	Public, Community & Population Health/Santé publique, santé communautaire et santé des populations
**Title / Titre:**	Ensuring a Successful Transition from Pap to HPV DNA Testing in Primary Cervical Cancer Screening: Exploring and Listening to Canadian Women's Needs is Critical for Effective Public Policy Change

Budget Recommendation/Recommandation budgétaire:

Reasonable. Both survey costs and research staff costs (which make up the lion's share of the budget) are reasonable.

**Review Type / Type d'évaluation:**	Reviewer 2 / Évaluateur 2
**Name of Applicant / Nom du chercheur:**	Rosberger, Zeev
**Application No. / Numéro de demande:**	420029
**Agency / Agence:**	CIHR/IRSC
**Competition / Concours:**	Project Grant/Subvention Projet
**Committee / Comité:**	Public, Community & Population Health/Santé publique, santé communautaire et santé des populations
**Title / Titre:**	Ensuring a Successful Transition from Pap to HPV DNA Testing in Primary Cervical Cancer Screening: Exploring and Listening to Canadian Women's Needs is Critical for Effective Public Policy Change

Please indicate your appraisal of the integration of sex as a biological variable as a strength, weakness, or not applicable to the proposal./Prière de sélectionner une option pour donner votre évaluation de l'intégration du sexe comme variable biologique en tant que point fort ou point faible de la proposition, ou en tant qu'élément non applicable à la proposition.

- (●) **Strength/Point fort**
- ( ) **Weakness/Point faible**
- ( ) **Not applicable/Non applicable**

Please indicate your appraisal of the integration of gender as a socio-cultural determinant of health as a strength, weakness, or not applicable to the proposal./Prière de sélectionner une option pour donner votre évaluation de l'intégration du genre comme déterminant socioculturel de la santé en tant que point fort ou point faible de la proposition, ou en tant qu'élément non applicable à la proposition.

- (●) **Strength/Point fort**
- ( ) **Weakness/Point faible**
- ( ) **Not applicable/Non applicable**

**Review Type / Type d'évaluation:**	Reviewer 2 / Évaluateur 2
**Name of Applicant / Nom du chercheur:**	Rosberger, Zeev
**Application No. / Numéro de demande:**	420029
**Agency / Agence:**	CIHR/IRSC
**Competition / Concours:**	Project Grant/Subvention Projet
**Committee / Comité:**	Public, Community & Population Health/Santé publique, santé communautaire et santé des populations
**Title / Titre:**	Ensuring a Successful Transition from Pap to HPV DNA Testing in Primary Cervical Cancer Screening: Exploring and Listening to Canadian Women's Needs is Critical for Effective Public Policy Change

Sex and/or Gender Considerations/Notions de sexe et/ou de genre:

The approach to this issue was thoughtful and intelligent. Cervical cancer is a disease of individuals of female sex, but issues of gender and sexuality are thoughtfully addressed throughout. Individuals eligible for participation are those with biological sex F at birth, regardless of gender or partner preference.

Hopefully the applicants can consider issues related to anal Pap and HPV testing (relevant to both sexes) in future work!

**Review Type / Type d'évaluation:**	Reviewer 3 / Évaluateur 3
**Name of Applicant / Nom du chercheur:**	Rosberger, Zeev
**Application No. / Numéro de demande:**	420029
**Agency / Agence:**	CIHR/IRSC
**Competition / Concours:**	Project Grant/Subvention Projet
**Committee / Comité:**	Public, Community & Population Health/Santé publique, santé communautaire et santé des populations
**Title / Titre:**	Ensuring a Successful Transition from Pap to HPV DNA Testing in Primary Cervical Cancer Screening: Exploring and Listening to Canadian Women's Needs is Critical for Effective Public Policy Change

Adjudication Criteria/Critères de sélection

Significance and Impact of the Research/Importance et impact de la recherche: 4.5

Approaches and Methods/Approches et méthodes: 4.0

Expertise, Experience and Resources/Expertise, expérience et ressources: 4.6

Top/Bottom Selection/Groupe supérieur/inférieur

- (●) **Top/Groupe supérieur**
- ( ) **Bottom/Groupe inférieur**

**Review Type / Type d'évaluation:**	Reviewer 3 / Évaluateur 3
**Name of Applicant / Nom du chercheur:**	Rosberger, Zeev
**Application No. / Numéro de demande:**	420029
**Agency / Agence:**	CIHR/IRSC
**Competition / Concours:**	Project Grant/Subvention Projet
**Committee / Comité:**	Public, Community & Population Health/Santé publique, santé communautaire et santé des populations
**Title / Titre:**	Ensuring a Successful Transition from Pap to HPV DNA Testing in Primary Cervical Cancer Screening: Exploring and Listening to Canadian Women's Needs is Critical for Effective Public Policy Change

Summary of Application/Résumé de la demande:

This proposed study aims to examine how Canadian women will adjust/adopt to the change in cervical screening practice. It proposes to use a web based cross sectional survey design to assess women's attitudes and beliefs about screening.

**Review Type / Type d'évaluation:**	Reviewer 3 / Évaluateur 3
**Name of Applicant / Nom du chercheur:**	Rosberger, Zeev
**Application No. / Numéro de demande:**	420029
**Agency / Agence:**	CIHR/IRSC
**Competition / Concours:**	Project Grant/Subvention Projet
**Committee / Comité:**	Public, Community & Population Health/Santé publique, santé communautaire et santé des populations
**Title / Titre:**	Ensuring a Successful Transition from Pap to HPV DNA Testing in Primary Cervical Cancer Screening: Exploring and Listening to Canadian Women's Needs is Critical for Effective Public Policy Change

Strengths and Weaknesses/Forces et faiblesses:

Strengths - large sample and proven health promotion frameworks, responded to previous reviews

Weakness - reliance on commercial business for cohort

**Review Type / Type d'évaluation:**	Reviewer 3 / Évaluateur 3
**Name of Applicant / Nom du chercheur:**	Rosberger, Zeev
**Application No. / Numéro de demande:**	420029
**Agency / Agence:**	CIHR/IRSC
**Competition / Concours:**	Project Grant/Subvention Projet
**Committee / Comité:**	Public, Community & Population Health/Santé publique, santé communautaire et santé des populations
**Title / Titre:**	Ensuring a Successful Transition from Pap to HPV DNA Testing in Primary Cervical Cancer Screening: Exploring and Listening to Canadian Women's Needs is Critical for Effective Public Policy Change

Budget Recommendation/Recommandation budgétaire:

Budget seems appropriate

Please indicate your appraisal of the integration of sex as a biological variable as a strength, weakness, or not applicable to the proposal./Prière de sélectionner une option pour donner votre évaluation de l'intégration du sexe comme variable biologique en tant que point fort ou point faible de la proposition, ou en tant qu'élément non applicable à la proposition.

- ○ **Strength/Point fort**
- ○ **Weakness/Point faible**
- ◉ **Not applicable/Non applicable**

Please indicate your appraisal of the integration of gender as a socio-cultural determinant of health as a strength, weakness, or not applicable to the proposal./Prière de sélectionner une option pour donner votre évaluation de l'intégration du genre comme déterminant socioculturel de la santé en tant que point fort ou point faible de la proposition, ou en tant qu'élément non applicable à la proposition.

- ○ **Strength/Point fort**
- ○ **Weakness/Point faible**
- ◉ **Not applicable/Non applicable**

**Review Type / Type d'évaluation:**	Reviewer 3 / Évaluateur 3
**Name of Applicant / Nom du chercheur:**	Rosberger, Zeev
**Application No. / Numéro de demande:**	420029
**Agency / Agence:**	CIHR/IRSC
**Competition / Concours:**	Project Grant/Subvention Projet
**Committee / Comité:**	Public, Community & Population Health/Santé publique, santé communautaire et santé des populations
**Title / Titre:**	Ensuring a Successful Transition from Pap to HPV DNA Testing in Primary Cervical Cancer Screening: Exploring and Listening to Canadian Women's Needs is Critical for Effective Public Policy Change

Sex and/or Gender Considerations/Notions de sexe et/ou de genre:
