# Supplementary material for: Ensuring a Successful Transition From Cytology to Human Papillomavirus–Based Primary Cervical Cancer Screening in Canada by Investigating the Psychosocial Correlates of Women’s Intentions: Protocol for an Observational Study
Source: JMIR Res Protoc. 2022 Jun 16;11(6):e38917. doi: 10.2196/38917 (PMC9247817; doi:10.2196/38917)
Supplement: Multimedia Appendix 4 [file resprot_v11i6e38917_app4.pdf]

Would you prefer to complete the survey in English or French? Préférez-vous répondre à ce questionnaire en anglais ou en français ?

a. English [>> PROCEED TO ENGLISH VERSION OF SURVEY]
a. Français [>> PROCEED TO FRENCH VERSION OF SURVEY; see page 20]

[Continue / Continuer]

Public health authorities are planning to introduce a new screening test to prevent cervical cancer in Canada. This survey is part of a research project funded by the Canadian government (through the Canadian Institutes of Health Research - CIHR) to understand Canadian women's attitudes, beliefs, and preferences about this upcoming change. <u>Your responses will be extremely helpful in deciding how to develop cervical cancer screening programs in the future.</u>

Please read carefully the following information about cervical cancer screening:

Cervical cancer is the fourth most common cancer in women worldwide. The **cervix** is the lower part of the uterus (womb). The cervix connects the uterus to the vagina (birth canal).

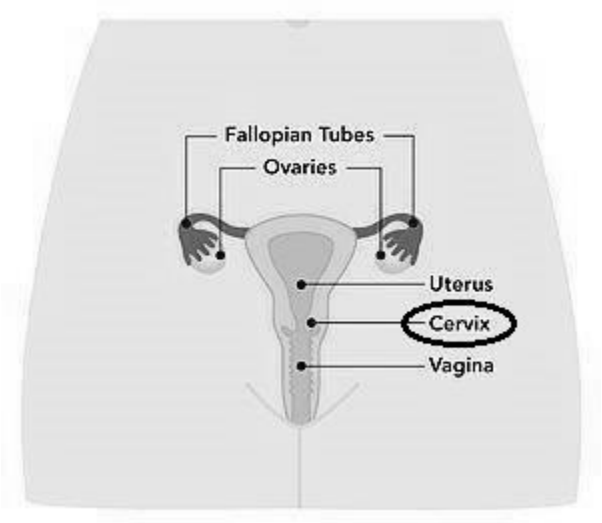

The **Pap test** (also called Pap smear) has been used to prevent cervical cancer for decades. During a **Pap tes**t, a healthcare professional uses a brush to gently scrape cells from your cervix. A new test called the **Human Papillomavirus (HPV) test** is also now available to prevent cervical cancer.

Assessing eligibility

1. How old are you?

   Enter age (in years) [TEXTBOX] [>>IF <21 OR >70, TERMINATE]

2. What is your biological sex (sex assigned at birth) as it appeared on your original birth certificate?
   a. Female
   b. Male [>> TERMINATE]
   c. I prefer not to answer

3. Do you have a cervix? (if your biological sex is female, the main reason for not having a cervix is if you have undergone surgery to remove the uterus, a "hysterectomy")
   a. Yes
   b. No [>> TERMINATE]
   c. Unsure [>> TERMINATE]

4. Have you ever been diagnosed with cervical cancer that required surgery, chemotherapy and/or radiation therapy?
   a. Yes [>> TERMINATE]
   b. No

[>>IF TERMINATED AT ANY POINT, DISPLAY:]

Thank you very much for taking the time to answer our questions. The answers we have received indicate that our survey with you is now completed. We thank you for your help.

Screening History and Health

1. How is your health in general?

    a. Excellent
    b. Very good
    c. Good
    d. Fair
    e. Poor
    f. Very poor

2. When did you have your last Pap test? Select the option that best describes you
    a. I had a Pap test within the last year
    b. I had a Pap test within the last 1 to 3 years [ANSWER A OR B = ADEQUATELY SCREENED]
    c. I had a Pap test over 3 years ago [>>ASK QUESTION 3]
    d. I have never had a Pap test [>>SKIP QUESTION 4, CONTINUE TO QUESTION 4] [ANSWER C OR D = UNDERSCREENED]

[>>IF ANSWER WAS C]

3. Did the COVID-19 pandemic prevent you from getting a Pap test?
    a. Yes
    b. No

[>>IF ANSWER TO QUESTION 2 WAS A, B, OR C]:

4. Have you received an abnormal Pap test result before?

    a. Yes
    b. No

5. Have you used oral birth control pills for 5 years or more?
    a. Yes
    b. No

6. To how many children have you given birth? [DROPDOWN MENU]
   0
   1
   2
   3
   4
   5
   6
   7
   8
   9
   10+

7. Which of the following best describes your tobacco smoking practices?
   a. I was never a smoker
   b. I smoked in the past but I do not smoke anymore
   c. I am currently a smoker

8. Have you received at least one dose of the HPV vaccine?
   a. Yes
   b. No
   c. I don't know

9. Do you have a family doctor?
   a. Yes
   b. No

10. Please choose your preferred type of measurement and enter your height and weight
    a. English (e.g., feet, pounds)

    i. Enter your height (ft + in.) [UPPER LIMIT 8ft]
    ii. Enter your weight (lbs) [UPPER LIMIT 700lbs]
  b. Metric (e.g., centimetres, kilograms)
    i. Enter your height (cm) [UPPER LIMIT 250cm]
    ii. Enter your weight (kg) [UPPER LIMIT 300kg]

11. Have you ever been diagnosed with any STI (sexually transmitted infection) (e.g. chlamydia, gonorrhea, HIV/AIDS, genital warts, etc)
    a. Yes
    b. No

12. Number of lifetime sexual partners
    a. 0 [>>SKIP QUESTION 14]
    b. 1-4
    c. 5-10
    d. More than 10
    e. Prefer not to answer

[IF ANSWER TO PREVIOUS QUESTION WAS B, C, D, OR E]:

13. How old were you (in years) when you first became sexually active with a partner?
    Pull down menu:
    - Before 12 years old
    - 12 years old
    - 13 years old
    - 14 years old
    - 15 years old
    - 16 years old
    - 17 years old
    - 18 years old
    - 19 years old

- 20 years old
- 21 years old
- 22 years old
- 23 years old
- 24 years old
- 25 years old
- 26 years old
- 27 years old
- 28 years old
- 29 years old
- 30+ years old
- Prefer not to answer

Cervical Cancer Knowledge

Please answer the following questions about cervical cancer and screening to the best of your ability. For each of the following, please indicate if the statement is 'true', 'false', or if you 'don't know'. All answers are important. [>>SELECT ONE ANSWER FOR EACH QUESTION] [SHOW QUESTIONS IN RANDOM ORDER]

	True	False	I don't know
1. A woman is at lower risk for developing cervical cancer if she smokes		X	
2. A woman is at higher risk of developing cervical cancer if she has had more than five sexual partners in her lifetime	X		
3. Vaginal bleeding between periods can be a sign of cervical cancer	X		
/ 14 items			

Human Papillomavirus (HPV) Testing Knowledge

Please answer the following questions about the HPV test to the best of your ability. For each of the following, please indicate if the statement is 'true', 'false', or if you 'don't know'. All answers are important. [>>SELECT ONE ANSWER FOR EACH QUESTION] [SHOW QUESTIONS IN RANDOM ORDER]

	True	False	I don't know
1. An HPV test can tell a woman how long she has had HPV		X	
2. An HPV test can be done at the same time as a Pap test	X		
3. If the HPV test shows that a woman has HPV, this means she is at increased risk for cervical cancer	X		
/ 14 items			

Please carefully read the following information about HPV testing:

Human papillomavirus (HPV) is the most common sexually transmitted infection. Almost all cervical cancers are caused by HPV. Cervical cancer is a highly preventable disease.

<u>**The Pap Test**</u>

- Cervical cells are looked at in the lab under a microscope to check for abnormal cells
- Currently used for routine cervical cancer screening in Canada
    - Every 2 or 3 years (starting at age 21 or 25, continuing until 65 or 70 depending on the province/territory in which you live)

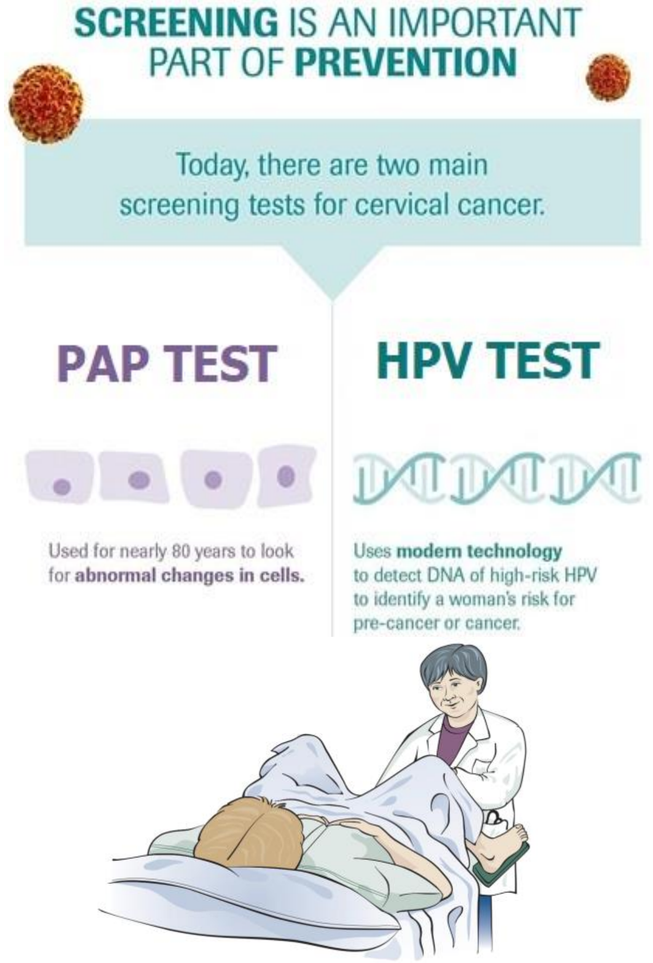

[Credit: CancerCare Manitoba]

<u>**The HPV Test**</u>

- Uses specialized technology to look for the presence of HPV DNA
- **Not yet used** for <u>routine</u> cervical cancer screening in Canada
    - Public health authorities are in the process of developing programs to introduce it
- Research shows that if HPV DNA is not found, women are at very low risk for cervical cancer and do not need to screen for cervical cancer as often as with the Pap test (e.g., every 5 years)

For both tests, the <u>procedure to collect the cell sample is the same</u> (see picture above)

Which of the following best describes where you are in your thinking about the **HPV test** as part of your routine cervical cancer screening?

a. At this moment, I have **not thought** about having the HPV test
b. At this moment, I am **undecided** about having the HPV test
c. At this moment, I **DO NOT** want to have the HPV test
d. At this moment, I **DO** want to have the HPV test
e. I **already had** the HPV test

Attitudes and Beliefs about HPV Testing

The following sections will present a series of statements about HPV testing. Please note that we are not asking about your knowledge. Whether or not you have had an HPV test in the past, we wish to know about your attitudes and beliefs. If you are unsure how to answer any question, simply select the answer that best reflects your attitudes and beliefs. [>>SELECT ONE ANSWER FOR EACH QUESTION] [SHOW QUESTIONS IN RANDOM ORDER]

Statement<br>I feel that …	Strongly disagree (1)	Disagree (2)	Somewhat disagree (3)	Neutral (4)	Somewhat agree (5)	Agree (6)	Strongly agree (7)
1. … I would be embarrassed to show my genitals to a healthcare professional during the HPV test							
2. … my partner's opinion about getting the HPV test would be important to me							
3. … having the HPV test would be a good way to identify problems before they become cancer							
4. … I would be worried about getting tested with the HPV test less often than every 3 years							
/ 44 items							

Please carefully read the following information about HPV self-sampling:

The HPV test detects the presence of HPV DNA in cervical cells. The collection of the cells for the HPV test can be done by your healthcare professional.

The collection of the cells can also be done by you in privacy. This is called **HPV self-sampling**, where you insert a thin swab into the vagina to collect cervical cells using a kit approved by Health Canada. The sample is then sent to a laboratory for analysis. The results are then returned to your clinic/healthcare provider, who will communicate with you. HPV self-sampling is **not yet used** but is being considered for routine cervical cancer screening in Canada. See the figure containing a description of HPV self-sampling in four steps.

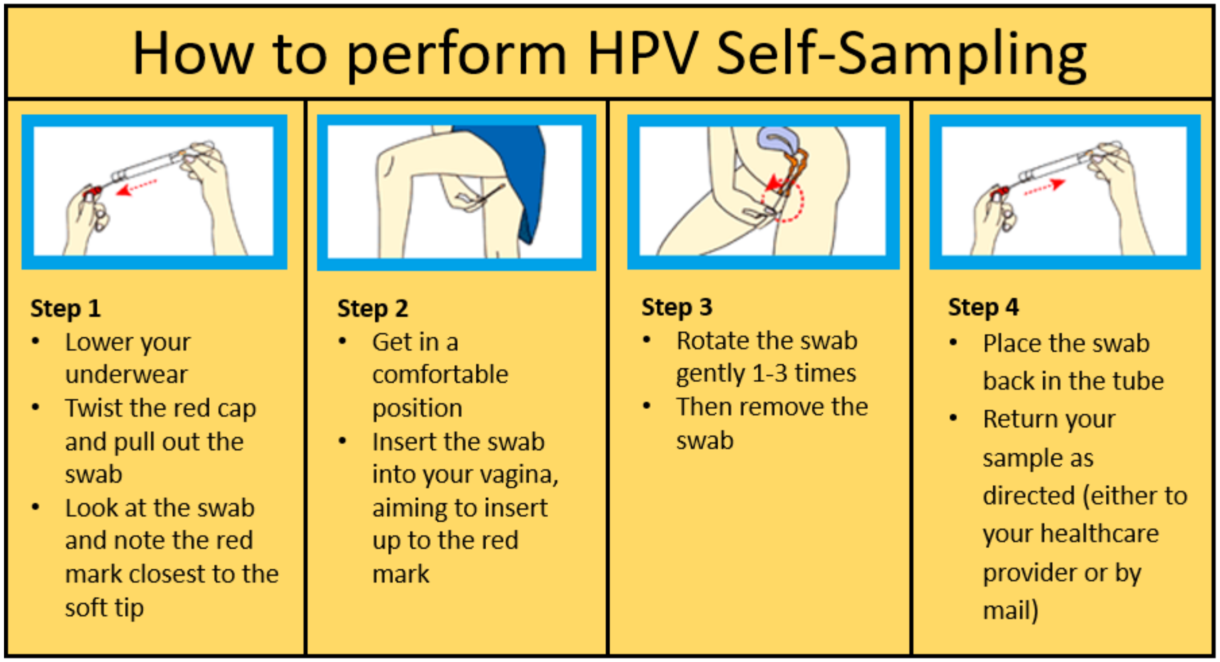

The following question is about HPV self-sampling.

Which of the following items best describes your thoughts about **HPV self-sampling** as part of your routine cervical cancer screening?

a. At this moment, I have **not thought** about doing HPV self-sampling
b. At this moment, I am **undecided** about doing HPV self-sampling
c. At this moment, I **DO NOT** want to do HPV self-sampling
d. At this moment, I **DO** want to do HPV self-sampling
e. I **already did** HPV self-sampling

Attitudes and Beliefs about HPV Self-sampling

The following sections will present a series of statements about HPV self-sampling. Please note that we are not asking about your knowledge. Whether or not you have done HPV self-sampling in the past, we wish to know your attitudes and beliefs. If you are unsure how to answer any question, simply select the answer that best reflects your attitudes and beliefs. [>>SELECT ONE ANSWER FOR EACH QUESTION] [SHOW QUESTIONS IN RANDOM ORDER]

Statement<br>I feel that…	Strongly disagree (1)	Disagree (2)	Somewhat disagree (3)	Neutral (4)	Somewhat agree (5)	Agree (6)	Strongly agree (7)
1. … if I did HPV self-sampling, I would worry that I am not doing it right							
2. … I would be more comfortable doing the swab by myself using HPV self-sampling than having an HPV test done by a healthcare professional							
/ 13 items							

Please carefully read the following information about cervical cancer screening options:

You have been presented with several methods for cervical cancer screening. Here is a reminder of what each of these methods are:

	Pap test	HPV test	
**Type of Analysis**	Cervical cells are looked at under a microscope to check for abnormal cells	Uses specialized technology to look for the presence of HPV DNA in cervical cells	
**Sample collection**	Done by a healthcare professional in a clinic/office	**<u>Option 1</u>** Done by a healthcare professional in a clinic/office	**<u>Option 2</u>** Self-sampling done by yourself in privacy using a kit approved by Health Canada

Imagine you are offered a choice among these methods by your healthcare professional. In this last set of questions, we want to know which of these methods you would prefer.

Preferences

In this next set of questions, you will be asked to select two out of four different situations: 1) **your least preferred** situation and 2) **your most preferred** situation. There should be two marks, one mark next to your most preferred situation, and one mark next to your least preferred situation.

Please see the sample question below:

Sample question		
LEAST preferred	Options	MOST preferred
X	I would prefer to watch movies once per week	
	I would prefer to play tennis two days per week	
	I would prefer to go shopping three times per week	
	I would prefer to go to the gym every day	X

In summary, you would have selected “I prefer to go to the gym every day” as your most preferred item, and “I prefer to watch movies once per week” as your least preferred item.

Please follow the same instructions for answering the next set of questions.

For each of the following nine questions, use the same strategy as in the example above to select **one** situation that you prefer **least** AND **one** situation that you prefer **most.** The questions may seem repetitive, but the last part of each sentence is different (where it says "every X years"). [SELECT ONE "LEAST PREFERRED" AND ONE "MOST PREFERRED" OPTION]

Question 1 (of 9)		
LEAST preferred	Options	MOST preferred
	Cervical cancer screening with the **Pap test** every 3 years	
	Cervical cancer screening with the **HPV test** every 5 years	
	Cervical cancer screening with **both the Pap test and the HPV test** every 10 years	
	Cervical cancer screening with the **HPV test using HPV self-sampling** every 5 years	

…

For each of the following nine questions, please select **one** statement that you prefer **least** AND **one** statement that you prefer **most.** The questions may seem repetitive, but the last part of each sentence is different (where it says "starting at X years old").

[SELECT ONE "LEAST PREFERRED" AND ONE "MOST PREFERRED" OPTION]

Question 1 (of 9)		
LEAST preferred	Options	MOST preferred
	Cervical cancer screening with the **Pap test** starting at age 21 years old	
	Cervical cancer screening with the **HPV test** starting at age 25 years old	
	Cervical cancer screening with **both the Pap test and the HPV test** starting at age 30 years old	
	Cervical cancer screening with the **HPV test using HPV self-sampling** starting at age 25 years old	

…

For the following question, please rank each option from 1 to 3, with 1 indicating your most preferred option and 3 your least preferred option.

[>>RANK OPTIONS 1-3]

1. I would prefer to receive information about cervical cancer and screening from:
    - Public health agency website (for example: Government of Canada, Health Canada, Canadian Partnership against cancer, etc.)
    - Social media e.g., Facebook, Instagram, Twitter
    - A healthcare professional (e.g, family physician, nurse, or gynaecologist)

For the following question, please rank each option from 1 to 4, with 1 indicating your most preferred option and 4 your least preferred option.

[>>RANK OPTIONS 1-4]

2. I would prefer to get the HPV test for my routine screening to prevent cervical cancer from:
    - Family physician
    - Gynaecologist
    - Nurse or nurse practitioner
    - Physician's assistant

Sociodemographics

1. Please select an option that best describe your ethnic origins.

    a. North American Aboriginal (First Nations, Inuit, Metis)
    b. Other North American (for example: Canadian, American, Ontarian, Quebecois, Acadian)
    c. European (for example: British, French, Western European, Eastern European)
    d. Caribbean (for example: Cuban, Haitian, Jamaican)
    e. Latin, Central and South American (for example: Mexican, Argentinian, Brazilian, Chilean)
    f. African (for example: Central and West African, North African, Southern African)
    g. Asian (for example: West Central Asian, South Asian, East and Southeast Asian)
    h. Oceania (Australian, New Zealander, Pacific Islander)
    i. Other
    Please specify: ________________ [TEXTBOX]

2. Do you identify as a visible minority?
    a. Yes
    b. No

3. My religious or spiritual belief system tends to influence my health decisions
    a. Yes
    b. No

4. What is the main language spoken in your home?
    a. English
    b. French
    c. Other language

5. Have you been living in Canada for the past 10 years or more?
    a. Yes
    b. No

6. Have you completed an apprenticeship or trades certificate/diploma, college or CEGEP degree, or a university degree?
    a. Yes
    b. No

7. Which gender do you identify as?
    a. Male
    b. Female
    c. Transgender Female/Trans Woman/Male-to-Female/MtF
    d. Transgender Male/Trans Man/Female-to-Male/FtM
    e. Genderqueer; neither exclusively male nor female
    f. Other
    Please specify: ____________________ [TEXTBOX]
    g. I prefer not to answer

8. How would you describe your present relationship/marital status?
    a. Married or living with a common law partner/spouse
    b. Single
    c. Dating or in a significant relationship with a partner you do not live with

9. Among the following categories, which one best reflects the annual total income, before taxes, of all the members of your household *before* the COVID-19 pandemic?
    a. $19 999 or less
    b. between $20 000 and $39 999
    c. between $40 000 and $59 999
    d. between $60 000 and $79 999
    e. between $80 000 and $99 999
    f. $100 000 or more
    g. I prefer not to answer

10. What was your employment status *in 2019*, *before* the COVID-19 pandemic?
    a. Employed Full time

b. Employed Part time
c. Not employed
d. Student
e. Retired
f. Caregiver (stay-at-home mother or caregiver for elderly parents, etc)
g. Other:
Please specify: ___________________ [TEXTBOX]

11. In what province or territory do you live? [DROPDOWN MENU]
Alberta
British Columbia
Manitoba
New Brunswick
Newfoundland and Labrador
Northwest Territories
Nova Scotia
Nunavut
Ontario
Prince Edward Island
Quebec
Saskatchewan
Yukon

12. Please estimate the travel time between your home and the nearest healthcare provider's office/clinic
a. Less than 30 min
b. 30-60 min
c. 60-120 min
d. More than 120 min

Closing page

Thank you very much for taking the time to answer our questions. The survey is now completed. We thank you for your help.

If you have any questions regarding this research study, you can contact the researcher in charge, Dr. Zeev Rosberger at (514) 340-8222 ext. 24215 or at zeev.rosberger@mcgill.ca or the study coordinator, Ben, at (514) 340-8222 ext. 23978 or at ben.haward@ladydavis.ca.

If you have questions or concerns about your health, please speak to a healthcare professional.

For all questions concerning your rights during your participation in this study, or if you have any complaints or comments regarding your experience in taking part in this research study, you can contact the Local Commissioner of Complaints and Quality of Service of the CIUSSS Centre-Ouest-de-l-Île-de-Montréal or the ombudsman of the institution at (514) 340-8222, ext. 24222.

If you would like more information about some of the topics brought up in this survey, you can visit these websites:

Additional information about HPV and cervical cancer:
https://hpvglobalaction.org/hpv-info/

https://www.cancer.ca/en/cancer-information/cancer-type/cervical/cervical-cancer/?region=on

Additional information about the HPV test:
https://www.cancer.ca/en/cancer-information/diagnosis-and-treatment/tests-and-procedures/hpv-test/?region=on

Canadian Provincial and Territorial Cervical Cancer Screening Programs or Recommendations

Province/ Territory	Start Age	Interval	Website
AB	25	3 years	https://screeningforlife.ca/cervical/
BC	25	3 years	http://www.bccancer.bc.ca/screening/cervix
MB	21	3 years	https://www.cancercare.mb.ca/screening/cervix

NB	21 or 3 years after becoming sexually active (whichever occurs later)	2-3 years; after 3 annual normal test results	https://www2.gnb.ca/content/gnb/en/departments/health/NewBrunswickCancerNetwork/content/ClinicalPracticeGuidelinesForCervicalCancerPreventionAndScreeningInNewBrunswick.html
NL	21	3 years, after 3 annual normal test results	https://cancercare.easternhealth.ca/prevention-and-screening/cervical-screening-program/
NS	25	3 years	https://library.nshealth.ca/Cancer/Screening#s-lg-box-15620653
NT	21 or 3 years after becoming sexually active (whichever occurs first)	2 years, after 3 annual normal test results	https://www.nthssa.ca/en/services/cancer-screening-programs/cervical-cancer-screening
ON	21	3 years	https://www.cancercareontario.ca/en/types-of-cancer/cervical/screening
PE	25	3 years	https://src.healthpei.ca/cervical-cancer-screening
QC	21	2-3 years	https://www.quebec.ca/en/health/advice-and-prevention/screening-and-carrier-testing-offer/cervical-cancer-screening
SK	21	3 years, after 3 biannual normal test results	http://www.saskcancer.ca/health-professionals-article/cancer-screening-guidelines-and-resources/cervical-cancer-screening
YT	N/A	N/A	N/A

Les autorités de santé publique envisagent de présenter un nouveau test de dépistage, voué à prévenir le cancer du col de l'utérus au Canada. Cette étude fait partie d'un projet de recherche financé par le gouvernement canadien, par l'intermédiaire des Instituts de recherche en santé du Canada (IRSC), afin de comprendre les croyances, l'opinion et les préférences des Canadiennes au sujet de ce changement à venir. Vos réponses contribueront à décider de la manière dont les programmes de dépistage du cancer du col de l'utérus seront développés à l'avenir.

Merci de lire attentivement les informations suivantes au sujet du dépistage du cancer du col de l'utérus :

Le cancer du col de l'utérus est le quatrième type de cancer le plus répandu chez les femmes dans le monde entier. Le col de l'utérus est la partie basse de l'utérus. Le col de l'utérus relie l'utérus au vagin.

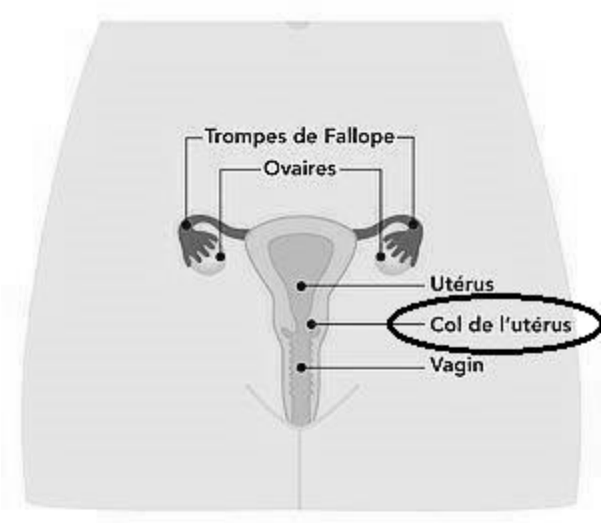

Le **Pap test** (également appelé frottis cervicovaginal) est utilisé depuis plusieurs décennies pour détecter un cancer du col de l'utérus. Lors d'un **Pap test**, un professionnel de la santé utilise une brosse pour gratter délicatement le col de l'utérus afin de prélever des cellules. Un nouveau test, le **test de dépistage du virus du papillome humain (VPH)** est à présent disponible pour prévenir le cancer du col de l'utérus.

Déterminer l'admissibilité

5. Quel âge avez-vous?

   Insérer âge [TEXTBOX] [>>IF <21 OR >70, TERMINATE]

6. Quel est votre sexe biologique (sexe assigné à la naissance), tel qu'inscrit sur votre certificat de naissance original?

   a. Femme
   b. Homme [>> TERMINATE]
   c. Je préfère ne pas répondre

7. Avez-vous un col de l'utérus? (si votre sexe biologique est féminin, la principale raison justifiant l'absence de col de l'utérus est l'hystérectomie, une intervention chirurgicale pour retirer l'utérus)
   a. Oui
   b. Non [>> TERMINATE]
   c. Je ne sais pas [>> TERMINATE]

8. Vous a-t-on déjà diagnostiqué un cancer du col de l'utérus nécessitant une chirurgie, une chimiothérapie et/ou une radiothérapie?
   a. Oui [>> TERMINATE]
   b. Non

[>>IF TERMINATED AT ANY POINT, DISPLAY:]

[Merci d'avoir pris le temps de répondre à nos questions. D'après les réponses que vous avez fournies, nous n'avons pas d'autres questions à vous poser pour cette enquête. Nous vous remercions pour votre aide.]

Historique de santé et dépistage

14. En général, comment est votre santé?

    a. Excellente
    b. Très bonne
    c. Bonne
    d. Passable
    e. Mauvaise
    f. Très mauvaise

15. Quand avez-vous fait pour la dernière fois Pap test? Choisissez l'option qui décrit le mieux votre cas.
    e. J'ai fait un Pap test au cours de la dernière année.
    f. J'ai fait un Pap test il y a plus de 1 an, mais moins de 3 ans [ANSWER A OR B = ADEQUATELY SCREENED]
    g. J'ai fait un Pap test il y a plus de 3 ans [>>ASK QUESTION 3]
    h. Je n'ai jamais fait de Pap test [>>SKIP QUESTION 3, CONTINUE TO QUESTION 4] [ANSWER C OR D = UNDERSCREENED]

[>>IF ANSWER WAS C]

16. Est-ce que la pandémie de la COVID-19 vous a empêché de réaliser un Pap test?
    a. Oui
    b. Non

[>>IF ANSWER TO QUESTION 2 WAS A, B, OR C]:

17. Avez-vous déjà reçu un résultat anormal de Pap test?

    a. Oui
    b. Non

18. Avez-vous utilisé une pilule contraceptive orale pendant 5 ans ou plus?
    a. Oui

b. Non

19. De combien d'enfants avez-vous accouché? [DROPDOWN MENU]
0
1
2
3
4
5
6
7
8
9
10+

20. Lequel de ces énoncés décrit le mieux vos habitudes en matière de cigarette?
    a. Je n'ai jamais fumé
    b. J'ai déjà fumé, mais ce n'est plus le cas actuellement
    c. Je fume actuellement

21. Avez-vous reçu au moins une dose du vaccin contre le VPH?
    a. Oui
    b. Non
    c. Je ne sais pas

22. Avez-vous un médecin de famille?
    a. Oui
    b. Non

23. Veuillez choisir votre type de mesure préféré et entrez votre taille et votre poids
    a. Anglais (ex. pieds, livres)
        i. Entrez votre taille (pi + po.) [UPPER LIMIT 8ft]
        ii. Entrez votre poids (lbs) [UPPER LIMIT 700lbs]
    b. Métrique (ex. centimètres, kilogrammes)
        i. Entrez votre taille (cm) [UPPER LIMIT 250cm]
        ii. Entrez votre poids (kg) [UPPER LIMIT 300kg]

24. Vous a-t-on déjà diagnostiqué une ITS (infection transmissible sexuellement); par exemple : chlamydia, gonorrhée, VIH/SIDA, verrues génitales, etc.?
    a. Oui
    b. Non

25. Nombre total de partenaires sexuels
    a. 0 [>>SKIP QUESTION 14]
    b. 1-4
    c. 5-10
    d. Plus de 10
    e. Je préfère ne pas répondre

[IF ANSWER TO PREVIOUS QUESTION WAS B, C, D, OR E]:

26. À quel âge êtes-vous devenue active sexuellement avec un·e partenaire?
    Menu déroulant
    - Avant 12 ans
    - 12 ans
    - 13 ans
    - 14 ans
    - 15 ans
    - 16 ans
    - 17 ans
    - 18 ans

- 19 ans
- 20 ans
- 21 ans
- 22 ans
- 23 ans
- 24 ans
- 25 ans
- 26 ans
- 27 ans
- 28 ans
- 29 ans
- 30 ans ou plus
- Je préfère ne pas répondre

Connaissances sur le cancer du col de l'utérus

Veuillez répondre du mieux que vous pouvez aux questions suivantes sur le cancer du col de l'utérus et son dépistage. Pour chaque énoncé, veuillez indiquer « vrai », « faux » ou « je ne sais pas ». Toutes les réponses sont importantes. [>>SELECT ONE ANSWER FOR EACH QUESTION] [SHOW QUESTIONS IN RANDOM ORDER]

	Vrai	Faux	Je ne sais pas
1. Une femme est moins à risque de développer le cancer du col de l'utérus si elle fume		X	
2. Une femme est plus à risque de développer le cancer du col de l'utérus si elle a eu plus de cinq partenaires sexuels	X		
3. Des saignements vaginaux en dehors des règles peuvent être un signe du cancer du col de l'utérus	X		
/ 14 items			

Connaissances sur le test du virus du papillome humain (VPH)

Veuillez répondre du mieux que vous pouvez aux questions suivantes sur le test du VPH. Pour chaque énoncé, veuillez indiquer « vrai », « faux » ou « je ne sais pas ». Toutes les réponses sont importantes. [>>SELECT ONE ANSWER FOR EACH QUESTION] [SHOW QUESTIONS IN RANDOM ORDER]

	Vrai	Faux	Je ne sais pas
1. Un test du VPH peut révéler depuis combien de temps une femme a le VPH		X	
2. Un test du VPH peut être réalisé en même temps qu'un Pap test	X		
3. Si le test du VPH révèle qu'une femme a le VPH, cela signifie qu'elle est plus à risque d'avoir un cancer du col de l'utérus	X		
/ 14 items			

Veuillez lire attentivement les informations suivantes sur le test du VPH :

Le virus du papillome humain (VPH) est l'infection transmissible sexuellement la plus courante. La quasi-totalité des cancers du col de l'utérus sont causés par le VPH. Le cancer du col de l'utérus est une maladie facilement évitable.

<u>Le Pap test</u>

- Les cellules cervicales sont examinées au microscope en laboratoire afin de détecter la présence de cellules anormales
- Est actuellement utilisé pour le dépistage de routine du cancer du col de l'utérus au Canada
  - Se fait tous les 2 ou 3 ans (à partir de l'âge de 21 ou 25 ans, jusqu'à 65 ou 70 ans, selon votre province ou territoire de résidence)

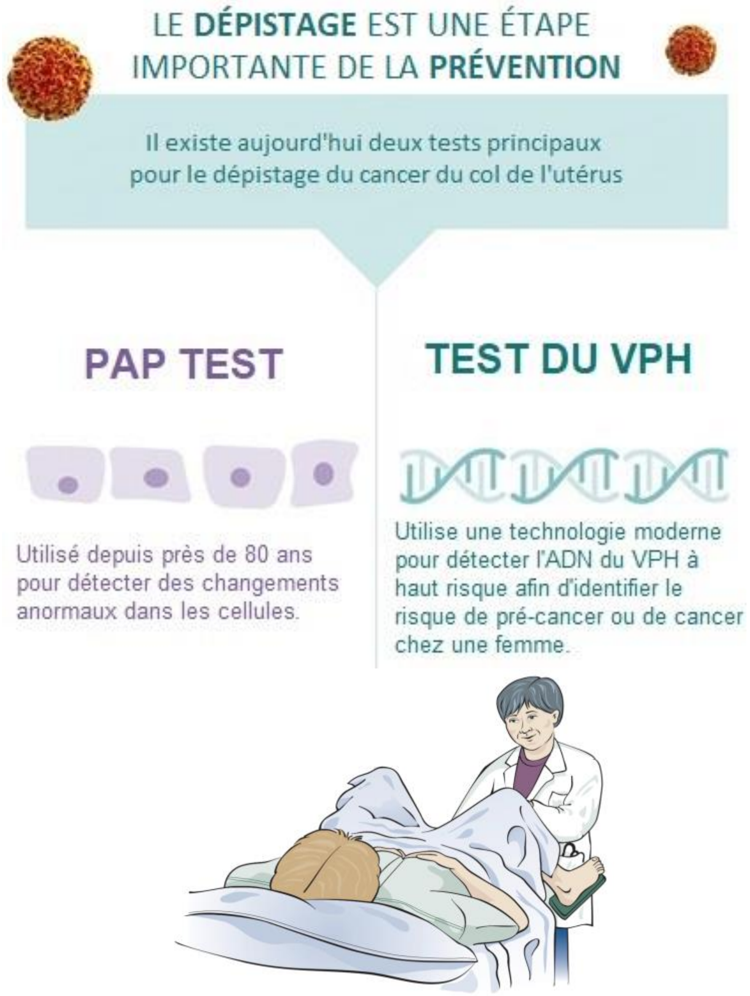

Pour les deux tests, la procédure de prélèvement d'échantillon de cellules est la même (voir le diagramme ci-dessus).

<u>Le test du VPH</u>

- Utilise une technologie spécialisée pour détecter la présence d'ADN de VPH
- **N'est pas** encore utilisé pour le dépistage <u>de routine</u> du cancer du col de l'utérus au Canada
  - Les autorités de santé publique développent actuellement des programmes pour l'introduire
- Les recherches montrent que si l'ADN de VPH n'est pas détecté, les femmes ont un très faible risque de développer un cancer du col de l'utérus et n'ont pas besoin de se faire dépister aussi souvent qu'avec un Pap test (par ex. tous les 5 années)

Lequel de ces énoncés décrit le mieux votre état d'esprit quant au **test du VPH** dans le cadre de votre dépistage de routine du cancer du col de l'utérus?

a. Pour le moment, je n'ai **pas pensé** au fait de réaliser le test du VPH
b. Pour le moment, je n'ai **pas pris de décision** sur le test du VPH
c. Pour le moment, je **NE veux PAS** réaliser le test du VPH
d. Pour le moment, je **VEUX** réaliser le test du VPH
e. J'ai **déjà réalisé** le test du VPH

Attitudes et opinions relatives au test du VPH

Les sections suivantes présentent une série d'énoncés sur le test du VPH. Veuillez noter que nous n'évaluons pas vos connaissances. Que vous ayez fait ou non le test du VPH par le passé, nous aimerions connaître votre attitudes et opinions. Si vous ne savez pas comment répondre à l'une de ces questions, veuillez simplement sélectionner la réponse qui reflète le plus votre attitudes et opinions.
[>>SELECT ONE ANSWER FOR EACH QUESTION] [SHOW QUESTIONS IN RANDOM ORDER]

	Je ne suis pas du tout d'accord	Je ne suis pas d'accord	Je ne suis pas vraiment d'accord	Neutre	Je suis plutôt d'accord	Je suis d'accord	Je suis entièrement d'accord
Je pense que...	(1)	(2)	(3)	(4)	(5)	(6)	(7)
1. … cela m'embarrasserait de montrer mes parties génitales à un professionnel de la santé lors d'un test du VPH							
2. … l'opinion de mon partenaire sur le fait de réaliser le test du VPH serait importante pour moi							
3. … faire le test du VPH serait un bon moyen d'identifier un problème avant que cela ne se transforme en cancer							
4. … cela m'inquiéterait de faire le test du VPH moins souvent qu'à tous les 3 ans							
/ 44 items							

Lequel de ces énoncés décrit le mieux votre état d'esprit quant au **test du VPH** dans le cadre de votre dépistage de routine du cancer du col de l'utérus?

a. Pour le moment, je n'ai **pas pensé** au fait de réaliser le test du VPH
b. Pour le moment, je n'ai **pas pris de décision** sur le test du VPH
c. Pour le moment, je **NE veux PAS** réaliser le test du VPH
d. Pour le moment, je **VEUX** réaliser le test du VPH
e. J'ai **déjà réalisé** le test du VPH

Veuillez lire attentivement les informations suivantes sur l’auto-prélèvement du test du VPH :

Le test du VPH détecte la présence d’ADN de VPH dans les cellules cervicales. Le prélèvement des cellules pour le test du VPH peut être réalisé par votre professionnel de la santé.

Vous pouvez également effectuer ce prélèvement par vous-même, en privé. C’est ce qu’on appelle **l’auto-prélèvement du test du VPH**, qui consiste à insérer une petite brosse fine dans le vagin afin de prélever des cellules cervicales, à l’aide d’une trousse approuvée par Santé Canada. L’échantillon est ensuite envoyé en laboratoire afin d’être analysé. Les résultats sont envoyés à votre professionnel de la santé, qui entrera en contact avec vous. L’auto-prélèvement du test du VPH **n’est pas encore** utilisé mais il est envisagé pour le dépistage <u>de routine</u> du cancer du col de l’utérus au Canada. Vous trouverez ci-joint une description de l’auto-prélèvement du test du VPH en quatre étapes.

Comment faire l’auto-prélèvement du test du VPH			
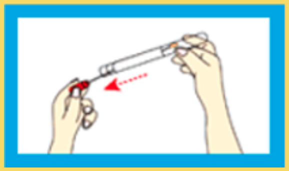<br>**Étape 1**<br>• Baissez votre sous-vêtement<br>• Tournez le capuchon rouge et retirez la brosse du tube<br>• Regardez la brosse et remarquez la marque rouge la plus proche de la pointe en coton	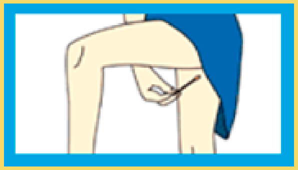<br>**Étape 2**<br>• Placez-vous dans une position confortable<br>• Insérez la brosse à l’intérieur de votre vagin jusqu’à la marque rouge	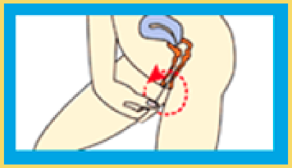<br>**Étape 3**<br>• Pivotez doucement la brosse 1 à 3 fois<br>• Ensuite, retirez la brosse	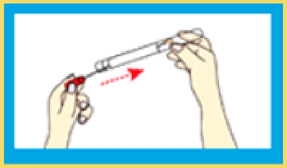<br>**Étape 4**<br>• Replacez la brosse a l’intérieur du tube<br>• Renvoyez cet échantillon selon les instructions (à votre médecin directement ou par la poste)

La question suivante concerne l'auto-prélèvement du test du VPH.

Lequel des énoncés suivants décrit le mieux votre état d'esprit quant à **l'auto-prélèvement** du test du VPH dans le cadre de votre dépistage de routine du cancer du col de l'utérus?

f. Pour le moment, je n'ai **pas pensé** au fait de faire un auto-prélèvement du test du VPH
g. Pour le moment, je n'ai **pas pris de décision** sur le fait de faire l'auto-prélèvement du test du VPH
h. Pour le moment, je **NE veux PAS** faire d'auto-prélèvement du test du VPH
i. Pour le moment, je **VEUX** faire un auto-prélèvement du test du VPH
j. J'ai **déjà fait** un auto-prélèvement du test du VPH

Attitudes et opinions relatives à l'auto-prélèvement du test du VPH

[SHOW ON A PAGE BEFORE AND ABOVE CAROSEL SCALES]: Les sections suivantes présentent une série d'énoncés sur l'auto-prélèvement du test du VPH. Veuillez noter que nous n'évaluons pas vos connaissances. Que vous ayez réalisé ou non l'auto-prélèvement du test du VPH par le passé, nous aimerions connaître votre attitudes et opinions. Si vous ne savez pas comment répondre à l'une de ces questions, veuillez simplement sélectionner la réponse qui reflète le plus votre attitudes et opinions. [>>SELECT ONE ANSWER FOR EACH QUESTION] [SHOW QUESTIONS IN RANDOM ORDER]

	Je ne suis pas du tout d'accord	Je ne suis pas d'accord	Je ne suis pas vraiment d'accord	Neutre	Je suis plutôt d'accord	Je suis d'accord	Je suis entièrement d'accord
Je pense que...	(1)	(2)	(3)	(4)	(5)	(6)	(7)
1. … si je faisais l'auto-prélèvement du test du VPH, j'aurais peur de ne pas le faire correctement							
6. … je serais plus à l'aise à l'idée de faire le prélèvement moi-même à l'aide de l'auto- prélèvement du test du VPH plutôt que de faire réaliser le test par un professionnel de la santé							
/ 13 items							

La question suivante concerne l'auto-prélèvement du test du VPH.

Lequel des énoncés suivants décrit le mieux votre état d'esprit quant à **l'auto-prélèvement** du test du VPH dans le cadre de votre dépistage de routine du cancer du col de l'utérus?

a. Pour le moment, je n'ai **pas pensé** au fait de faire un auto-prélèvement du test du VPH
b. Pour le moment, je n'ai **pas pris de décision** sur le fait de faire l'auto-prélèvement du test du VPH
c. Pour le moment, je **NE veux PAS** faire d'auto-prélèvement du test du VPH
d. Pour le moment, je **VEUX** faire un auto-prélèvement du test du VPH
e. J'ai **déjà fait** un auto-prélèvement du test du VPH

Merci de lire attentivement les informations suivantes au sujet des différentes options de dépistage du cancer du col de l'utérus :

Nous vous avons présenté plusieurs méthodes de dépistage du cancer du col de l'utérus. Voici un rappel de ces différentes méthodes :

	**Le Pap test**	**Le test du VPH**	
**Type d'analyse**	Les cellules cervicales sont regardées au microscope afin de détecter la présence de cellules anormales	Utilise une technologie spécialisée pour détecter la présence d'ADN de VPH	
**Prélèvement de l'échantillon**	Elle est réalisée par un professionnel de la santé dans une clinique ou cabinet médical	**Option 1**<br>Elle est réalisée par un professionnel de la santé dans une clinique ou cabinet médical	**Option 2**<br>Le prélèvement des cellules peut être effectué par vous-même en privé

Imaginez que l'on vous propose plusieurs options pour le dépistage du cancer du col de l'utérus par votre professionnel de la santé. Dans cette dernière série de questions, nous aimerions savoir quelles options vous préférez.

Préférences

Dans la série de questions qui suit, vous devrez choisir deux des quatre situations : 1) la situation **que vous préférez le moins** et 2) la situation **que vous préférez le plus.** Il devrait y avoir deux crochets, un à côté de la situation que vous préférez le plus, et un à côté de la situation que vous préférez le moins.

Veuillez-vous référer à l'exemple suivant :

Exemple de question		
Je préfère le MOINS	Options	Je préfère le PLUS
X	Je préférerais regarder des films une fois par semaine	
	Je préférerais jouer au tennis deux jours par semaine	
	Je préférerais aller magasiner trois fois par semaine	
	Je préférerais aller à la salle de sport tous les jours	X

En résumé, vous auriez choisi « Je préférerais aller à la salle de sport tous les jours » comme situation que vous aimez le plus, et « Je préférerais regarder des films une fois par semaine » comme situation que vous aimez le moins.

Veuillez suivre les mêmes instructions pour répondre à la série de questions qui suit.

Pour chacune des neuf prochaines questions, utilisez la méthode illustrée dans l'exemple ci-dessus afin de choisir une situation que vous préférez **le moins** ET une situation que vous préférez **le plus.** Les questions peuvent sembler répétitives, mais la dernière partie des phrases change (où il est écrit « tous les X ans »).[SELECT ONE "LEAST PREFERRED" AND ONE "MOST PREFERRED" OPTION] [DISPLAY "tous les X ans" IN BLUE]

Question 1 (of 9)		
Je préfère le MOINS	Options	Je préfère le PLUS
	Un dépistage du cancer du col de l'utérus à l'aide d'un **Pap test** tous les 3 ans	
	Un dépistage du cancer du col de l'utérus à l'aide d'un **test du VPH** tous les 5 ans	
	Un dépistage du cancer du col de l'utérus à l'aide d'un **Pap test et d'un test du VPH** tous les 10 ans	
	Un dépistage du cancer du col de l'utérus à l'aide de **l'auto-prélèvement du test du VPH** tous les 5 ans	

Pour chacune des neuf prochaines questions, utilisez la méthode illustrée dans l'exemple ci-dessus afin de choisir une situation que vous préférez **le moins** ET une situation que vous préférez **le plus.** Les questions peuvent sembler répétitives, mais la dernière partie des phrases change (où il est écrit « tous les X ans »).

Question 1 (of 9)		
Je préfère le MOINS	Options	Je préfère le PLUS
	Un dépistage du cancer du col de l'utérus à l'aide d'un **Pap test** dès l'âge de 21 ans	
	Un dépistage du cancer du col de l'utérus à l'aide d'un **test du VPH** dès l'âge de 25 ans	
	Un dépistage du cancer du col de l'utérus à l'aide d'un **Pap test et d'un test du VPH** dès l'âge de 30 ans	
	Un dépistage du cancer du col de l'utérus à l'aide de **l'auto-prélèvement du test du VPH** dès l'âge de 25 ans	

Pour la question suivante, veuillez classer chaque option de 1 à 3, où 1 indique l’option que vous préférez le plus et 5 indique l'option que vous préférez le moins.
[>>RANK OPTIONS 1-3]

1. Je préférerais recevoir des informations au sujet du cancer du col de l’utérus et du dépistage de la part de:
   - Site Web d’une agence de santé publique (par exemple : le Gouvernement du Canada, Santé Canada, Partenariat canadien contre le cancer, etc.)
   - Réseaux sociaux, par exemple Facebook, Instagram, Twitter
   - Professionnel de la santé

Pour la question suivante, veuillez classer chaque option de 1 à 4, où 1 indique l’option que vous préférez le plus et 5 indique l'option que vous préférez le moins.
[>>RANK OPTIONS 1-4]

2. Je préférerais faire réaliser le test du VPH dans le cadre de mon dépistage de routine du cancer du col de l’utérus par:
   - Mon médecin de famille
   - Un gynécologue
   - Un/une infirmier/ère ou infirmier/ère praticien/ne
   - Un adjoint au médecin

Données socio-démographiques

1. Sélectionnez une option qui décrit le mieux vos origines ethniques parmi les options suivantes. [SELECT ONE]
    a. Nord-américain autochtone (Premières Nations, Inuits, Métis)
    b. Autre nord-américain (par exemple: canadien, américain, ontarien, québécois, acadien)
    c. Européen (par exemple: britannique, français, européen de l'ouest, européen de l'est)
    d. Caraïbes (par exemple: cubain, haïtien, jamaïcain)
    e. Amérique latine, centrale et sud-américaine (par exemple: mexicain, argentin, brésilien, chilien)
    f. Africain (par exemple: Afrique centrale et occidentale, Afrique du Nord, Afrique australe)
    g. Asiatique (par exemple: Asie centrale occidentale, Asie du Sud, Asie de l'Est et du Sud-Est)
    h. Océanie (australienne, néo-zélandaise, insulaire du Pacifique)
    i. Autre (veuillez préciser) : ________________________ [TEXTBOX]

2. Vous considerez-vous membre d'une minorité visible?
    a. Oui
    b. Non

3. Mes croyances religieuses ou spirituelles ont tendance à influencer mes décisions concernant ma santé
    a. Oui
    b. Non

4. Quelle est la langue principalement parlée chez vous?
    a. Anglais
    b. Français
    c. Autre langue

5. Vivez-vous au Canada depuis 10 ans ou plus?
    a. Oui
    b. Non

6. Avez-vous obtenu un certificat/diplôme d'apprentissage ou d'une école de métier, un diplôme de collège ou de cégep, ou un diplôme universitaire?
    a. Oui
    b. Non

7. À quel genre vous identifiez-vous?

    a. Homme
    b. Femme
    c. Femme transgenre/Femme trans/Transition homme-femme/MtF
    d. Homme transgenre/Homme trans/Transition femme-homme/FtM
    e. De genre queer; ni exclusivement homme ou femme
    f. Autre (veuillez préciser) : ____________________ [TEXTBOX]
    g. Je préfère ne pas répondre

8. Comment décririez-vous votre état matrimonial/civil actuel?
    a. Marié.e ou vivant avec un.e conjoint.e de fait ou époux/épouse
    b. Célibataire
    c. En couple ou en relation avec un.e partenaire ne vivant pas dans votre foyer

9. Parmi les catégories suivantes, laquelle reflète le mieux le revenu total annuel avant impôts de tous les membres de votre foyer *avant* la pandémie de la COVID-19?
    a. 19 999 $ ou moins
    b. entre 20 000 $ et 39 999 $
    c. entre 40 000 $ et 59 999 $
    d. entre 60 000 $ et 79 999 $
    e. entre 80 000 $ et 99 999 $
    f. 100 000 $ ou plus
    g. Je préfère ne pas répondre

10. Quelle était votre situation professionnelle *en 2019,* avant la pandémie de COVID-19?

a. Emploi à temps plein
a. Emploi à temps partiel
c. Sans emploi
d. Aux études
e. À la retraite
f. Proche aidant (parent au foyer ou proche aidant pour des parents âgés, etc.)
Autre : Veuillez préciser : __________________ [TEXTBOX]

11. Dans quelle province ou quel territoire résidez-vous? [DROPDOWN MENU]
Alberta
Colombie-Britannique
Île-du-Prince-Édouard
Manitoba
Nouveau-Brunswick
Nouvelle-Écosse
Nunavut
Ontario
Québec
Saskatchewan
Territoires du Nord-Ouest
Terre-Neuve-et-Labrador
Yukon

12. Veuillez fournir le temps de trajet approximatif entre votre foyer et la clinique ou le cabinet médical le plus proche
a. Moins de 30 minutes
b. 30-60 minutes
c. 60-120 minutes
d. Plus de 120 minutes

Page de fermeture

Merci beaucoup d'avoir pris le temps de répondre à nos questions. Ce questionnaire est maintenant terminé. Nous vous remercions pour votre aide.

Si vous avez des questions sur ce projet de recherche, vous pouvez contacter le chercheur responsable, Dr Zeev Rosberger au (514) 340-8222 poste 24215, ou à l'adresse zeev.rosberger@mcgill.ca, ou le coordinateur de l'étude, Ben Haward, au (514) 340-8222 poste 23978, ou à l'adresse ben.haward@ladydavis.ca

Si vous avez des questions ou des inquiétudes quant à votre santé, parlez-en à un professionnel de la santé.

Pour toute question concernant vos droits pendant votre participation à cette étude, ou si vous avez des plaintes ou des commentaires au sujet de votre expérience de participation à cette étude de recherche, vous pouvez contacter le commissaire aux plaintes et à la qualité des services du CIUSSS du Centre-Ouest-de-l'Île-de-Montréal ou l'ombudsman de l'institution au (514) 340-8222, poste 24222.

Si vous souhaitez obtenir plus d'informations sur les sujets évoqués dans cette enquête, vous pouvez consulter les sites Web suivants :

Informations complémentaires au sujet du VPH et du cancer du col de l'utérus :

https://vphactionglobale.org/hpv-info-fr/

https://www.cancer.ca/fr-ca/cancer-information/cancer-type/cervical/cervical-cancer/?region=on

Informations complémentaires au sujet du test du VPH :

https://www.cancer.ca/fr-ca/cancer-information/diagnosis-and-treatment/tests-and-procedures/hpv-test/?region=on

Programmes de dépistage du cancer du col de l'utérus provinciaux/territoriaux au Canada

Province/ Territoire	Début	Intervalle	Site Web
AB	25 ans	3 ans	https://screeningforlife.ca/cervical/
BC	25 ans	3 ans	http://www.bccancer.bc.ca/screening/cervix

MB	21 ans	3 ans	https://www.cancercare.mb.ca/screening/cervix
NB	21 ans ou 3 ans après le début de l'activité sexuelle	2-3 ans, après 3 résultats de tests annuels normaux	https://www2.gnb.ca/content/gnb/fr/ministeres/sante/reseau_du_cancer_du_nouveau-brunswick/content/Guide_de_pratique_clinique_pour_la_prevention_et_depistage_du_cancer_du_col_uterin_au_Nouveau-Brunswick.html
NL	21 ans	3 ans, après 3 résultats de tests annuels normaux	https://cancercare.easternhealth.ca/prevention-and-screening/cervical-screening-program/
NS	25 ans	3 ans	https://library.nshealth.ca/Cancer/Screening#s-lg-box-15620653
NT	21 ans ou 3 ans après le début de l'activité sexuelle	2 ans, après 3 résultats de tests annuels normaux	https://www.nthssa.ca/fr/services/cancer-screening-programs/d%C3%A9pistage-du-cancer-du-col-de-l%E2%80%99ut%C3%A9rus
ON	21 ans	3 ans	https://www.cancercareontario.ca/fr/types-de-cancer/cancer-du-col-de-uterus/depistage
PE	25 ans	3 ans	https://src.healthpei.ca/cervical-cancer-screening
QC	21 ans	2-3 ans	https://www.quebec.ca/sante/conseils-et-prevention/depistage-et-offre-de-tests-de-porteur/depistage-du-cancer-du-col-uterus
SK	21 ans	3 ans, après 3 résultats de tests biannuels normaux	http://www.saskcancer.ca/health-professionals-article/cancer-screening-guidelines-and-resources/cervical-cancer-screening
YT	N/A	N/A	N/A
